# Development of a high-throughput screening platform for identification of functional BACH1 inhibitors reveals compounds with anti-invasive potential

**DOI:** 10.1016/j.redox.2026.104187

**Published:** 2026-04-24

**Authors:** Kevin X. Ali, Donika Klenja-Skudrinja, Maureen Higgins, David Walker, Yumna Sharaf, Martin Dankis, Angana A.H. Patel, Dorota Raj, Jozefina J. Dzanan, Esben B. Svenningsen, Alistair Langlands, Thomas Poulsen, Tadashi Honda, Albena T. Dinkova-Kostova, Clotilde Wiel, Volkan I. Sayin, Laureano de la Vega

**Affiliations:** a Institute of Clinical Sciences, Department of Surgery, Sahlgrenska Center for Cancer Research, University of Gothenburg, Gothenburg, Sweden; b Wallenberg Centre for Molecular and Translational Medicine, University of Gothenburg, Gothenburg, Sweden; c Jacqui Wood Cancer Centre, Division of Cancer Research, School of Medicine, University of Dundee, UK; d Department of Chemistry, Aarhus University, Denmark; e National Phenotypic Screening Centre, School of Life Sciences, University of Dundee, UK; f Department of Chemistry and Institute of Chemical Biology & Drug Discovery, Stony Brook University, Stony Brook, NY, USA

**Keywords:** BACH1, Drug screening, Functional Inhibitors, Anti-metastatic

## Abstract

BACH1 is a transcriptional regulator that modulates various cytoprotective pathways. Among these pathways BACH1 regulates cellular oxidative stress responses by suppressing the expression of cytoprotective genes. Dysregulated BACH1 activity has been implicated in a range of pathologies, including chronic inflammatory diseases, fibrosis, and cancer, making it a promising therapeutic target. However, BACH1 remains an underexploited drug target, with limited pharmacological inhibitors available.

We have developed a novel luciferase-based reporter cell line enabling quantitative, high-throughput assessment of BACH1 inhibition. Using this platform, we rigorously screened two small-molecule libraries with 2046 compounds and identified four structurally distinct compounds that robustly inhibit BACH1 function. Notably, these compounds also activate transcription factor NRF2, suggesting the potential for a broader modulation of oxidative stress pathways.

Importantly, we demonstrate that commonly used 2D migration assays may fail to detect phenotypes consistent with BACH1 inhibition, resulting in false negatives. In contrast, we establish that 3D invasion assays more robustly capture anti-invasive effects of BACH1 functional inhibition. Using this 3D system, we validate the identified compounds as potent suppressors of lung cancer cell invasion *in vitro*.

This study delivers a novel screening platform for BACH1-targeted drug discovery, and challenges current *in vitro* standards by establishing 3D invasion assays as a more accurate functional readout for BACH1-targeting compounds. Additionally, it identifies new dual functional BACH1 inhibitors/NRF2 activators, offering novel chemical scaffolds for the development of anti-metastatic therapies and potentially treatments for diseases driven by oxidative stress and inflammation.

## 1 Background

BTB and CNC homology 1 (BACH1) is a member of the Cap ‘n' Collar and basic region leucine zipper family (CNC-bZip) of transcription factors. It is widely expressed in mammalian tissues and is involved in the regulation of oxidative stress and inflammatory responses, metabolic pathways related to heme and iron, ferroptosis, and cancer cell migration and invasion [[1], [2], [3], [4], [5], [6], [7], [8], [9], [10], [11], [12]]. The best characterised and most consistently regulated BACH1 target gene is the enzyme heme oxygenase 1, encoded by *HMOX1,* which catalyses the rate-limiting reaction in heme degradation and has important antioxidant and anti-inflammatory properties [[12], [13], [14], [15], [16], [17]]. Given its regulatory role across diverse pathways, BACH1 has emerged as an attractive therapeutic target for a wide range of diseases including cancer, Parkinson's disease, sickle cell disease, ischemia/reperfusion injury, non-alcoholic steatohepatitis, insulin resistance, coronary artery disease, myocardial infarction and tuberculosis [2,[17], [18], [19], [20], [21], [22], [23], [24], [25], [26], [27], [28], [29]].

Another key CNC-bZip transcription factor related to, and in some cases competing with, BACH1 is NRF2 (Nuclear factor erythroid 2-related factor 2). NRF2 binds to Antioxidant Response Elements (ARE) in the promoters of its target genes and triggers their expression. A subset of NRF2 target genes is also regulated by BACH1. However, while NRF2 induces the expression of genes that protect against oxidative stress and inflammation and suppress ferroptosis, BACH1 represses them [1,10,11,16,30].

In homeostatic conditions, NRF2 binds to its negative regulator Kelch-like ECH-associated protein 1 (KEAP1) and is targeted for ubiquitination and subsequent proteasomal degradation [31,32]. Most compounds that stabilise NRF2 are either electrophiles that covalently modify specific cysteine residues in KEAP1, impairing its activity [33,34], or protein–protein interaction inhibitors that block NRF2-KEAP1 binding [35,36]. In contrast, known BACH1-targeting compounds act by reducing BACH1 nuclear levels, either by regulating its localisation (i.e. inducing its nuclear export) and/or its stability [21,24,[37], [38], [39], [40], [41]] (i.e. reducing BACH1 levels). Thus, these compounds are not classical biochemical inhibitors, as they do not affect BACH1 activity *per se*.

Both NRF2 activation and BACH1 inactivation have protective effects in numerous experimental models of disease driven by oxidative stress or inflammation [22]. While NRF2 broadly activates cytoprotective genes, BACH1 inhibition triggers a more restricted response but with a strong upregulation of *HMOX1* (significantly stronger than the one obtained upon NRF2 activation) [[37], [38], [39],^42^], making *HMOX1* induction a sensitive marker for BACH1 suppression. As such, combined NRF2 activation and BACH1 inactivation is expected to produce a more potent antioxidant and anti-inflammatory effect than targeting either factor alone, potentially resulting in improved therapeutic efficacy against a variety of chronic conditions, as shown for age-related neurodegeneration [43]. Moreover, BACH1 regulates unique targets not shared with NRF2 and can dominantly repress genes even in the presence of active NRF2 [42]. This confers BACH1 inhibition distinct therapeutic value, particularly in contexts such as cancer cell invasion, where its suppression yields anti-metastatic effects [37,38]. Interestingly, we have previously shown that some NRF2 activators also inhibit BACH1 [38], suggesting that dual-activity compounds may be more common than currently recognised. Despite their promise, few BACH1 or dual BACH1/KEAP1 inhibitors have been reported, highlighting the need for novel therapeutic candidates.

To address this need, we leveraged the strong correlation between *HMOX1* induction and BACH1 inhibition [37,39,42]. Because in our previous analyses *HMOX1* was the only BACH1 target gene consistently and significantly regulated across multiple cell lines [42], we generated a reporter cell line in which *HMOX1* is fused to luciferase, enabling functional screening for BACH1 inhibitors. Using this cell line, we screened two compound libraries and identified and validated four novel BACH1 functional inhibitors. Notably, all four compounds have inhibitory activity against BACH1 function while also activating NRF2. These findings confirm the utility of this screening platform for identifying or repurposing compounds with therapeutic potential in conditions involving oxidative stress, inflammation, and metastasis.

## 2 Materials & methods

### 2.1 Cell lines and reagents

Cells were cultured in Roswell Park Memorial Institute 1640 (RPMI1640) Medium (HaCaT) or Dulbecco's Modified Eagle Medium (DMEM) (H1299, A549, MDA-MB-231, KP and KPK cells) at 37 °C and 5% CO2. Media were obtained from Thermo Fisher Scientific and supplemented with 10% FBS. All cells were either validated by Short Tandem Repeat (STR) profiling or obtained from ATCC and were routinely tested for mycoplasma. KP (*Kras*^*LSL-G12D/+*^*; p53*^*flox/flox*^) and KPK (KEAP1-KO, *Kras*^*LSL-G12D/+*^*; p53*^*flox/flox*^) cells were previously derived from mouse lung tumours as described [44]. CRISPR-edited BACH1-KO cells were produced as previously described [37,38].

Antibodies against BACH1 (F-9) and LAMIN B2 (C-20) were obtained from Santa Cruz Biotechnology (sc-271211 and sc-56147), and against NRF2 were obtained from Cell Signalling (12721) and Abcam (ab62352). Antibody against ALPHA-TUBULIN was obtained from Sigma-Aldrich (T5168). Horseradish peroxidase (HRP)-conjugated secondary antibodies were from Life Technologies. Information for all the compounds used is provided in Supplementary Table S1. TBE31 and TBE56 were synthesised as described [45,46]. All siRNAs used were OnTargetplus SMARTPool siRNAs obtained from Horizon Discovery.

### 2.2 Generation of HMOX1-Luc cells

**Fig. 1:**
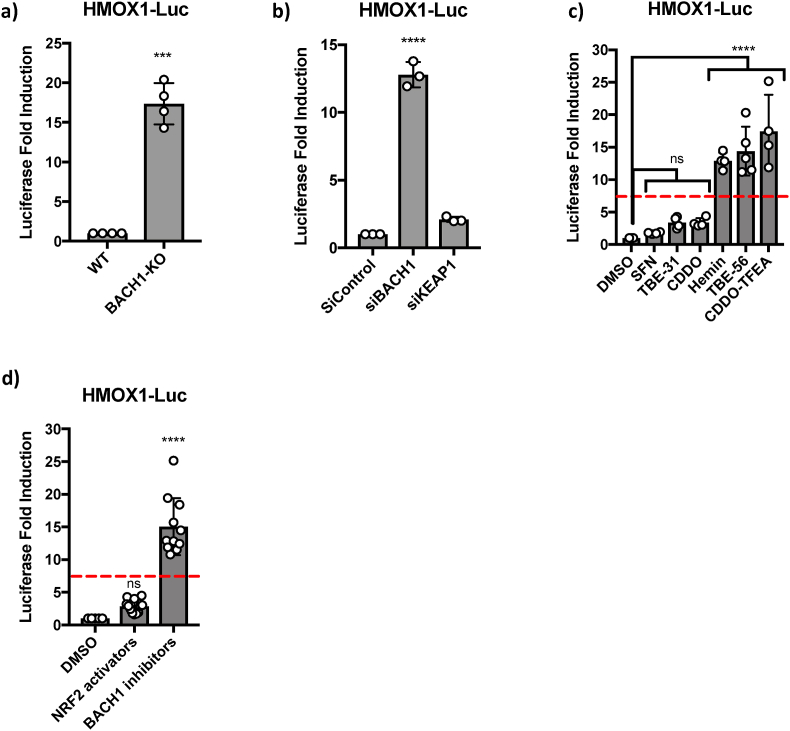
**Validation of H1299 HMOX1-Luc cells as BACH1 reporter cells. a)** The levels of basal luciferase in H1299 HMOX1-Luc WT and BACH1-KO were compared. Data (n = 4) represent means ± SD and are expressed relative to the WT sample. **b)** H1299 HMOX1-Luc cells were transfected with either siRNA control (siControl), siRNA against BACH1 (siBACH1) or an siRNA against KEAP1 (siKEAP1). 48 h later their luciferase levels were measured. Data (n = 3) represent means ± SD and are expressed relative to the siControl sample. **c)** H1299 HMOX1-Luc cells were treated with SFN (5 μM), TBE31 (100 nM), CDDO (100 nM), hemin (10 μM), TBE56 (100 nM) or CDDO-TFEA (100 nM). 16 h later luciferase was measured. Data (n = 4-5) represent means ± SD and are expressed relative to the DMSO treated sample; red lane indicates a selected threshold (7.5-fold) that can be used to differentiate between KEAP1 inhibitors (NRF2 activators) and BACH1 inhibitors. **d)** As in 1c but with all the single data from NRF2 activators or BACH1 inhibitors pulled together.

To generate H1299 HMOX1-Luc, CRISPR-Cas9 together with a gRNA located at the stop codon of HMOX1 (GCTTTATGCCATGTGAATGC) was used to knock-in the luciferase gene into the endogenous HMOX1 locus. The stop codon was removed and GFP and luciferase were inserted, using the T2A and P2A sequences between them. GFP was used to select for positive cells. After sequence validation, a selected clone was validated functionally (see Fig. 1).

### 2.3 Luciferase assay

The H1299 HMOX1 reporter cells were seeded at 80,000 cells per well in 150 μl of DMEM +10% FCS in 96 well plates (Thermo Scientific™ Nunc MicroWell Optical-Bottom Plate with Polymer Base). The next day, compounds were added to the cells (3 technical replicates per compound). 16 h later, 100 μl of media was removed from all wells and 50 μl of reconstituted Bright Glo Luciferase Assay Substrate (Promega, USA) was added. The plate was incubated at room temperature (RT) while shaking at 130 rpm for 5 min before measuring the luminescence using a plate reader (Infinite F Plex Tecan).

### 2.4 Library composition

The CLOUD library [47] was designed to cover all therapeutically significant space of FDA-approved drugs within a format compatible with a single 384 well plate. The library contains 263 compounds, including prodrugs and active metabolites, selected for their chemical diversity. The Tocriscreen Plus (and add-on) library, is a combination of the first iteration of the Tocriscreen library (1280 compounds), plus the compounds which were added to Tocriscreen 2.0 (503 additional compounds); this provides 1783 bio-active compounds, with a broad range of pharmacological targets (>300), including kinases, non-kinase enzymes, ion channels, nuclear receptors, 7TM receptors, transporters, and cell biology-related targets. A complete list of the screened compounds from these libraries is included in Supplementary File 2.

### 2.5 384 well plate assay

Actively growing H1299 HMOX1-Luciferase cells were transferred into 384-well plates (Greiner μClear, 781091) at densities displayed in figures, or at 1.4x10^5^ cells/cm^2^ (15,000 cells/well) for screening experiments, and in 50 μl per well, using a Multidrop Combi (ThermoFisher). One column on each 384-well plate contained media only, serving as a blank on each assay plate. Cells were allowed to settle for 15 min at room temperature (RT), and incubated for 24h at 37 °C, 5% CO_2_. Compounds were added using an Echo 550 (Beckmann Coulter), and cells incubated for 16h at 37 °C, 5% CO_2_. Three columns on each assay plate were reserved for control treatments, meaning each control was present in 16 wells on each assay plate: one column of negative control (DMSO), one column of positive control (10 μM hemin), and one column of a non-specific inducer (100 nM CDDO). Test compounds from the CLOUD (Enamine, 263 compounds on one assay plate) and Tocriscreen (Tocris, 1280 compounds from Tocriscreen 1.0 over 4 assay plates and an additional 503 compounds from Tocriscreen 2.0 over 2 assay plates) libraries were dispensed into random wells to a final concentration of 10 μM. Assay plates and Bright-Glo Luciferase were equilibrated to RT, 20 μl media per well was aspirated (leaving 30 μl media) using a 405LS (Agilent), and 30 μl Bright-Glo Luciferase was added per well using a Tempest (Formulatrix). The reaction was incubated for 5 min at RT, 300 rpm on a ThermoMixer C (Eppendorf), then the luminescence was measured on an EnSpire (PerkinElmer). Blank-corrected luminescence was calculated, and the value for each well was divided by the median of the DMSO controls on each plate to obtain plate-normalised fold change values. The blank-corrected luminescence values for hemin- and DMSO-treated wells were used to calculate the rZ’ ($1-\left(\frac{3*\left(MAD\left(+ve\;ctrl\right)+MAD\left(-ve\;ctrl\right)\right)}{Median\;\left(+ve\;ctrl\right)-Median\;\left(-ve\;ctrl\right)}\right)$) and SB ($\frac{Mean\left(+ve\;ctrl\right)}{Mean\left(-ve\;ctrl\right)}$) values for each plate.

### 2.6 siRNA cell transfections

On the day prior to transfection, cells were plated to the required cell density (70-90% confluency). Lipofectamine RNAiMAX (Invitrogen) was used to introduce the siRNA. The siRNA and lipofectamine were individually diluted in OptiMEM (Gibco) and incubated for 10 min at RT. Diluted siRNA was added to the diluted Lipofectamine solution (1:1 ratio) and further incubated for 15 min. The siRNA-lipid complex was added to the cells and incubated 48h in a humidified incubator at 37 °C and 5% CO_2_ before harvesting.

### 2.7 Quantitative real time PCR (rt-qPCR)

RNA was extracted using GeneJET RNA Purification Kit (Thermo Fisher Scientific) and 500 ng of RNA per sample was reverse-transcribed to cDNA using Omniscript RT kit (Qiagen) supplemented with RNase inhibitor according to the manufacturer's instructions. The resulting cDNA was processed using TaqMan Universal Master Mix II (Life Technologies, Carlsbad, CA, USA) as well as corresponding Taqman probes. Gene expression was determined using a QuantStudio 7 Flex qPCR machine by the comparative ΔΔCt method. All experiments were performed between three and seven times, and data were normalised to the housekeeping gene HPRT1.

### 2.8 Cell lysis and Western blot

Cells were washed and harvested in ice-cold phosphate-buffered saline (PBS). For whole-cell extracts, cells were lysed in RIPA buffer supplemented with phosphate and protease inhibitors. Lysates were sonicated for 20 s at 20% amplitude and then subjected to centrifugation for 10 min at 4 °C. For subcellular fractionation, cells were resuspended in 400 μl of low-salt buffer A (10 mM HEPES/KOH pH7.9, 10 mM KCL, 0.1 mM EDTA, 0.1 mM EGTA, 1 mM β-mercaptoethanol) and incubated for 10 min on ice before 10 μl of 10% NP-40 was added followed by gently vortexing. The homogenate was subjected to centrifugation for 1 min at 13,000 rpm in a microcentrifuge, and the supernatant representing the cytoplasmic fraction was collected. The pellet containing the cell nuclei was washed 4 additional times in buffer A then resuspended in 100 μl high-salt buffer B (20 mM Hepes/KOH pH7.9, 400 mM NaCl, 1 mM EDTA, 1 mM EGTA, 1 mM β-mercaptoethanol). The resuspended fractions were sonicated followed by centrifugation at 4 °C for 10 min at 13,000 rpm. The supernatant representing the nuclear fraction was then collected and saved. The protein concentration in the whole cell lysate, nuclear and cytoplasmic fractions were determined using the BCA assay (Thermo Fisher Scientific, Waltham, MA, USA). The lysates were then mixed with SDS sample buffer and boiled for 5 min at 95 °C. Equal amounts of protein from lysates were separated by SDS-PAGE, followed by semidry blotting to a polyvinylidene difluoride membrane (PVDF) (Thermo Fisher Scientific). Subsequently, the membrane was blocked with 5% (w/v) non-fat dried milk dissolved in Tris-buffered saline (TBS) with 0.1% v/v Tween-20 (TBST), followed by incubation with primary antibodies overnight at 4 °C. The next day, the membranes were washed with TBST and appropriate secondary antibodies coupled to HRP were added and incubated for 1h at RT. Following another set of TBST washes, the bound secondary antibodies were detected by enhanced chemiluminescence using ClarityTM Western ECL Blotting Substrate (Bio-Rad, Hercules, CA, USA). The resulting protein bands were quantified and normalised to each lane's loading control using ImageJ. For whole-cell extracts, the protein of interest was normalised against TUBULIN. LAMIN was used as an internal control for nuclear fractions and TUBULIN was used as control for cytoplasmic fractions.

### 2.9 Cell viability assay

Alamar Blue (Thermo Fisher Scientific) was used to determine cell viability after drug treatment. Cells were seeded in 96-well plates to reach 50–60% confluency and were treated the next day with the corresponding compounds. After 48h, the treatment was removed and replaced with Alamar Blue diluted in the corresponding growth medium (1:10 ratio). After 6h of incubation at 37 °C the fluorescence was measured (excitation at 535 nm and emission at 590 nm) using a microplate reader (Infinite F Plex Tecan). The viability was calculated relative to the DMSO-treated control.

### 2.10 Transwell migration assay

The transwell migration assay was conducted using Corning™ Transwell™ Multiple Well Plate with 6.5 mm inserts and 8.0 μm pore permeable polyester membrane (Fisher Scientific). Cells were trypsinised followed by resuspension in growth medium containing 10% FBS. The cells were then washed in PBS and subsequently resuspended in serum-free media. 70,000–100,000 cells, suspended in 150 μl serum-free media, supplemented with the drug of choice, were seeded on the upper chamber of the insert and 600 μl of complete media (10% FBS v/v) (supplemented with the drug of choice) was added to the bottom well.

After 16h, the transwells were washed in PBS before using 4% PFA to fix the migrated cells for 15 min, followed by another PBS wash and finally staining with crystal violet (0.025%) for 20 min. The crystal violet was then aspirated, and the non-migrated cells remained in the upper chamber were removed using a cotton swab wet in PBS. The transwells were washed to remove excess crystal violet by dipping them a few times in a beaker filled with distilled water. Imaging of the transwells was conducted on five different areas of the well under bright-field microscope (20x). Images were analysed blind to the experimental conditions, and stained cells were automatically counted using ImageJ. The mean cell count was derived from five images per well and normalised to respective controls.

### 2.11 3D spheroid collagen invasion assay

For 3D spheroid collagen invasion assays, cells were seeded at 20,000 cells per well in ultra-low attachment 96-well plates (Corning™ 96-Well Clear Ultra Low Attachment Microplates, 7007) and incubated for 48h to allow spheroid formation. Spheroids were then embedded in a collagen I matrix together with the compounds at the indicated concentrations. After 24h the media was exchanged with fresh media containing the same compound concentration. Spheroids were incubated for 48h post-embedding. Spheroid invasion was assessed using two complementary approaches. First, spheroid area was monitored using the Incucyte 5SX Live Imaging System. Spheroid area within collagen was quantified using the Incucyte Spheroid Analysis Module and normalised to the area of non-embedded spheroids imaged in parallel to determine collagen-dependent invasion.

Second, Images were acquired using a 10× objective under a bright-field microscope, and the number of sprouts and invasion area per spheroid was manually quantified using ImageJ.

## 3 Results

*Generation of a BACH1-reporter cell line:* To generate a suitable reporter cell line for identifying BACH1 inhibitors, we selected the human lung adenocarcinoma cell line H1299. This is an adherent cell line which is easy to culture and maintain, with a doubling time of around 25h, high levels of BACH1, low levels of NRF2 (it harbours an active KEAP1), and robust responses to both BACH1 and NRF2 modulation [42]. We used CRISPR-based gene editing to create a H1299 *HMOX1-Luc* reporter cell line by introducing the coding sequence of luciferase under the control of the endogenous *HMOX1* promoter *(See M&M)*.

We next tested whether luciferase induction faithfully reported BACH1 downregulation by measuring the effect of BACH1 depletion on luciferase levels. The luciferase activity was significantly and greatly induced in response to both CRISPR- and siRNA-mediated BACH1 depletion (Fig. 1a and b and Suppl. Fig. S1a), but was not significantly affected by KEAP1 depletion (Fig. 1b and Suppl. Fig. S1a). These results validate this cell line as a BACH1-responsive reporter cell line and confirm that KEAP1 depletion (and the consequent NRF2 stabilisation) is not sufficient to induce robust *HMOX1* expression in our setting. Once we confirmed the response of the cell line to genetic BACH1 depletion, we optimised the reporter assay in a medium-throughput setting (96-wells) and examined luciferase induction in response to the BACH1 inhibitors hemin and TBE56 [37], the dual BACH1/KEAP1 inhibitor CDDO-TFEA [38], and to the NRF2 activators SFN, TBE31 and CDDO, which inhibit KEAP1 but not BACH1. Fig. 1c and d shows that the luciferase reporter cell line responds well to the various BACH1 inhibitors and can discriminate between BACH1 inhibitors and compounds that inhibit KEAP1 alone (NRF2 activators). Notably, although it is well established that NRF2 regulates the expression of *HMOX1*, we have previously shown that in many cell lines, NRF2 activation is a weak inducer of *HMOX1* [[37], [38], [39], [40]]; this is now confirmed in our assay using both genetic and pharmacological means for NRF2 activation.

*Optimisation of the screening assay in 384-well format*: The *HMOX1-Luc* assay was miniaturised to 384-well plate format through a series of optimisation experiments determining the effect of cell density and DMSO concentration on assay performance. To evaluate the quality and robustness of our screening assay, we calculated both the signal-to-background (SB) ratio and the robust Z′-factor (rZ′), two key parameters that assess assay reliability. A high SB ratio (>5) indicates a clear distinction between the signal of interest and the background noise, and the rZ′ factor is a statistical measure of assay quality, with values approaching 1.0 denoting increasing confidence in the assay's ability to differentiate positive and negative samples. In our assays, SB increased with cell density; however, increasing density above 1.1x10^5^ cells/cm^2^ (1.25x10^4^ cells/well) did not improve rZ’ (Suppl Fig. S2a, S2b and S2d), likely as a result of increased variability in hemin-treated wells at the higher densities. A blind spike-in experiment, in which hemin (BACH1 inhibitor) and CDDO (NRF2 activator) were transferred into random test wells, confirmed the ability of the assay to identify wells treated with compounds increasing *HMOX1*-luc activity (Suppl. Fig. S2b). Based on these results, the primary screen and dose response assays would be performed at a density of 15,000 cells/well (1.4x10^5^ cells/cm^2^). A DMSO tolerance experiment confirmed that the assay was robust to increasing concentrations of DMSO (up to 0.5%, Suppl. Fig. S2c): SB only slightly decreased above 0.3% DMSO; and although rZ′ was <0.5 at concentrations of DMSO ≥0.3%, this was likely due to increased variability by hemin treatment at these concentrations.

**Fig. 2:**
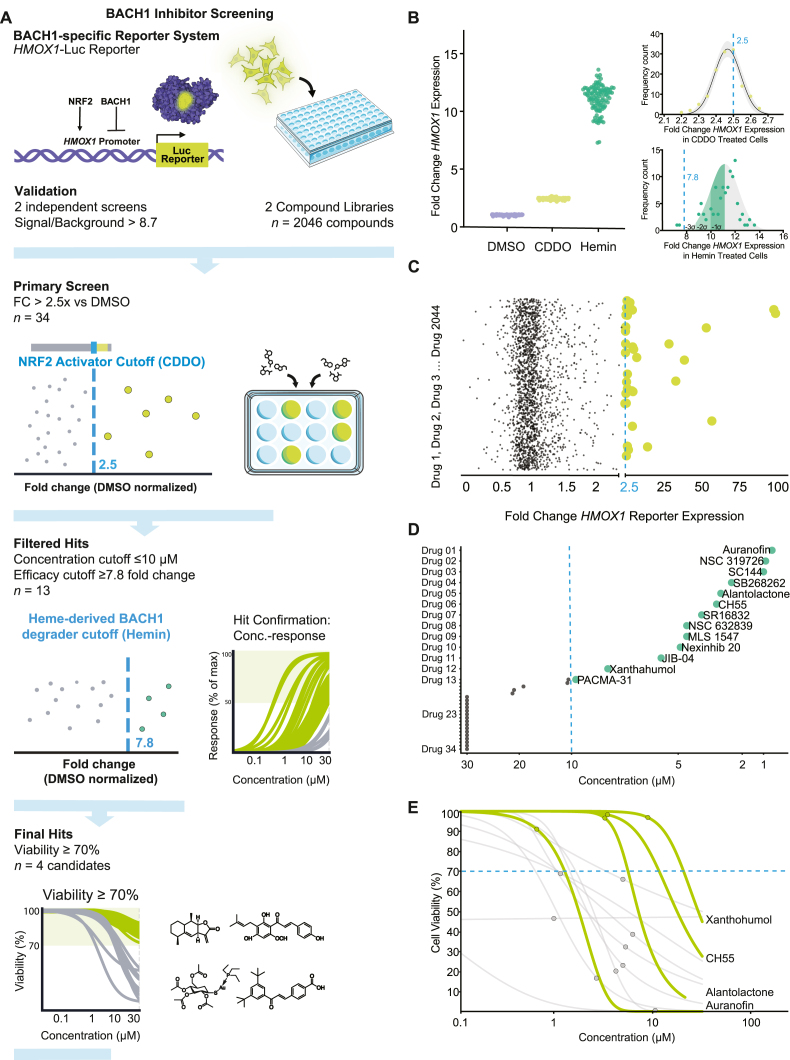
**High-throughput screening using an *HMOX1*-luciferase reporter system identifies small-molecule BACH1 inhibitors. a)** Schematic overview of the multistep drug screen; First step, *HMOX1*-Luc reporter assay used in two independent screens with a total of 2046 compounds. Second step, primary candidate selection at *HMOX1* expression greater than the NRF2 activator CDDO (cut-off), 34 hits. Third step, subsequent concentration-response investigation comparing the 34 selected compounds to the BACH1 inhibitor hemin (cut-off), 13 candidates. Fourth step, viability assays and selection at ≥70% viability at the previously identified concentration, resulted in four final candidates. **b)** HMOX1-Luc reporter assay performance. *Left panel*: distribution of fold-change (relative to DMSO) in luciferase activity for DMSO, the NRF2 activator CDDO and the BACH1 inhibitor hemin. *Right panel*: frequency distribution plots depicting the normal distribution 95% confidence interval band for CDDO (upper), 2.5-fold change cut-off depicted with dashed blue line, used as activation thresholds. Similarly, hemin-based cut-off (lower) at three standard deviations from mean (7.8-fold change). **c)** Primary screen of 2046 compounds (y-axis) investigated for fold change *HMOX1*-Luc expression (x-axis) and showing 34 compounds (green circles) which resulted in higher fold change than CDDO (2.5 FC). **d)** Concentration-response investigation data applying a concentration cut-off of ≤10 μM (using hemin as a reference) yielded 13 compounds that induced 7.8-fold HMOX1-Luc at concentrations below 10 μM (green circles). **e)** Cell-viability profiling across 0.1-30 μM concentration; cut-off for selected concentrations (circles) above 70% viability. Four compounds (alantolactone, auranofin, CH55, and xanthohumol) met both activity and viability criteria (green circles);. *Luciferase structure adapted from the RCSB PDB Molecule of the Month: Luciferase (*https://pdb101.rcsb.org/motm*) and 96-well and 12-well plate image adapted from Servier Medical Art (*https://smart.servier.com/*), licensed under CC BY 4.0 (*https://creativecommons.org/licenses/by/4.0/*).*

*Library screening*: Following confirmation that the 384-well plate-based luminescence assay was sufficiently robust for screening, we used the assay to screen the CLOUD (Enamine) and Tocriscreen (Tocris) compound libraries as a proof of concept to answer whether our reporter cell line can be used to identify novel BACH1 inhibitors. Fig. 2a illustrates the complete screening workflow, with summary panels for the corresponding results presented in Fig. 2b–e. The screen was performed as two independent replicates, with random compound placement used to locate the test compounds in different wells between replicates and to minimise the possibility of false positives. There was a clear separation in luminescence values between DMSO-, CDDO-, and hemin-treated wells (Fig. 2b and Suppl Fig. S2b, S2d and S2e), which was also apparent in the plate-normalised fold change values (Fig. 2b and Suppl. Fig. S2f). Assay performance was acceptable, with rZ’ > 0.5 and SB > 8.7, in all screening plates (Suppl. Fig. S2g). The majority of screened compounds did not increase the luminescence signal over the DMSO control (Fig. 2c and Suppl. Fig. S2e and S2f), but 70 test wells were identified where the fold change was >2.5, the mean fold change for CDDO-treated wells (Fig. 2b). Compound performance between the replicates was consistent (Suppl. Fig. S2h), showing a high level of correlation between the fold change values obtained for each compound in replicate 1 and replicate 2, indicating a likely low rate of false positives due to plate effects. Overall, there were 34 compounds identified with an average fold change greater than 2.5 from the two experimental replicates (Fig. 2a and c and Suppl. Fig. S2f and S2i). Compounds screening assays are typically performed at concentrations ranging between 1 and 10 μM [48]. In this study, both libraries were screened at a single concentration of 10 μM. This concentration provides a balance between achieving a higher hit rate than a 1 μM screen and avoiding the increased false positives, DMSO tolerance issues, and compound precipitation often observed at higher concentrations [49] (e.g., up to 100 μM). While single-dose screening may underestimate the total number of BACH1 inhibitory compounds, a 10 μM screening concentration can still identify compounds with activities of up to ∼40 μM [48]. Conversely, false negatives may occur for compounds with lower effective doses if toxicity is observed at 10 μM.

**Fig. 3:**
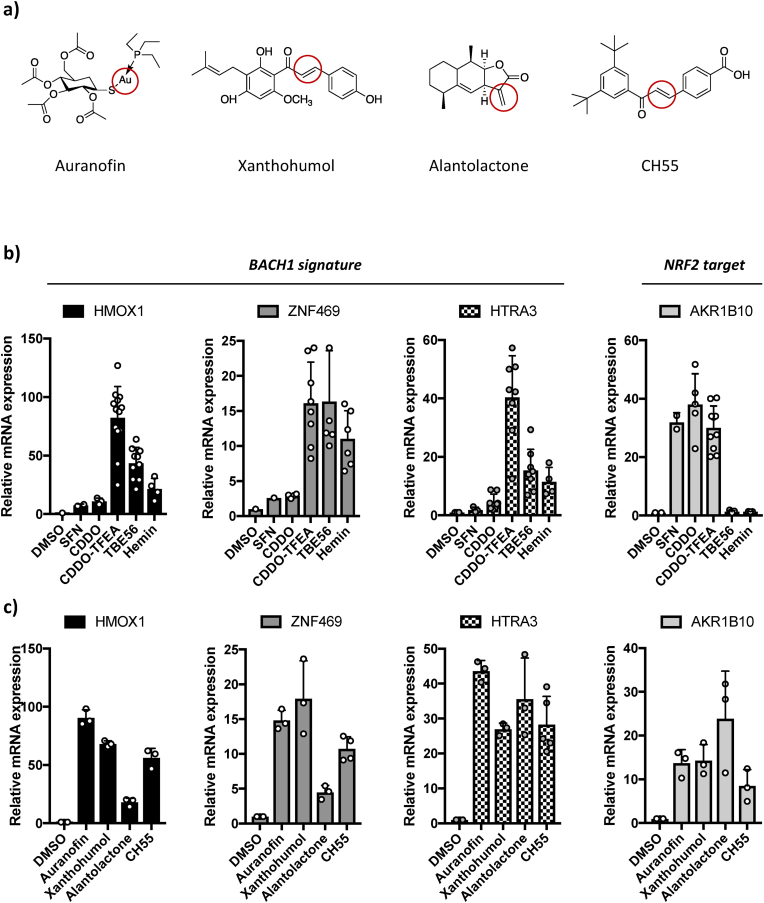
**Identified hits induce a BACH1 signature. a)** Chemical structure of the identified compounds; the electrophilic centre is circled in red. **b and c)** H1299 cells were treated with either b) DMSO, SFN (5 μM), CDDO (100 nM), CDDO-TFEA (100 nM), TBE56 (100 nM) and hemin (10 μM); or c) auranofin (0.5 μM), xanthohumol (10 μM), alantolactone (3 μM) or CH55 (5 μM). 16 h later, cells were lysed, and mRNA levels of the indicated genes were analysed by real-time qPCR. Data (a minimum of n = 3) represent means ± SD and are expressed relative to the DMSO treated sample.

The 34 hits from the primary screen were tested again in a concentration-response experiment, using 8 concentrations, ranging from 0.01 to 30 μM. *First selection criteria (efficacy)*: using the 7.8-fold induction of *HMOX1*-Luc achieved by 10 μM hemin as a benchmark, we selected compounds that were equally or more efficacious than hemin and removed any compound that needed more than 10 μM to reach this threshold (Fig. 2a and d and Suppl. Fig. S2j). This initial efficacy filter yielded 13 candidate compounds. *Second selection criteria (toxicity)*: while bona fide BACH1 inhibitors do not induce cell death at effective concentrations (those needed to inhibit BACH1) (Suppl. Fig. S2k), compounds that are cytotoxic or induce cellular stress may activate *HMOX1* through off-target mechanisms, potentially leading to false positives. To identify (and exclude) compounds that induce cell toxicity, we incubated H1299 cells with increasing concentrations of the selected compounds (from 0.1 μM up to 30 μM) over 48h and measured cell viability (Fig. 2a and e and Suppl. Fig. S2l). Compounds were excluded if the concentration required for 7.8-fold *HMOX1*-Luc induction resulted in <70% viability after 48h (arbitrary threshold). This stringent selection process yielded four candidates (auranofin, xanthohumol, alantolactone and CH55) (structures in Fig. 3a) which were selected for downstream validation.

*Compound validation:* To confirm the involvement of BACH1 in the compound-mediated induction of luciferase, we first tested the effect of the compounds in a BACH1 knockout (KO) HMOX1-Luc cell line. In the absence of BACH1, compound-induced HMOX1-luciferase activity was markedly reduced, indicating BACH1 dependence (Suppl. Fig. S3a). To exclude potential reporter- or cell line-specific artefacts, we next examined the effects of the compounds on endogenous *HMOX1* expression in the human keratinocyte HaCaT cell line. Consistent with the reporter data, the compounds robustly induced endogenous *HMOX1* expression in HaCaT cells (Supplementary Fig. S3b). Importantly, this induction was largely abolished by BACH1 depletion but not by NRF2 depletion, further supporting a central role for BACH1 mediating *HMOX1* induction in response to these compounds.

To further assess BACH1 involvement, we examined whether the identified compounds induced the expression of a lung cancer BACH1 gene signature we recently identified [42]. We used the induction of *HMOX1*, *ZNF469* and *HTRA3* expression as surrogate for BACH1 inhibition, and *AKR1B10* induction as a marker for NRF2 activation; control compounds included SFN (KEAP1 inhibitor), CDDO (KEAP1 inhibitor), CDDO-TFEA (dual KEAP1/BACH1 inhibitor [38]), TBE56 (BACH1 inhibitor [37]) and hemin (BACH1 inhibitor). As previously shown [42], the BACH1 signature genes are strongly induced upon BACH1 inhibition but not by NRF2 activation alone (Fig. 3b). At the concentrations tested, all four compounds identified in the screen were potent inducers of the three BACH1 target genes (Fig. 3c), with alantolactone showing a moderate *HMOX1* and *ZNF469* induction. All compounds also induced the expression of *AKR1B10*, consistent with NRF2 activation, suggesting they might act as both functional BACH1 inhibitors and NRF2 activators.

**Fig. 4:**
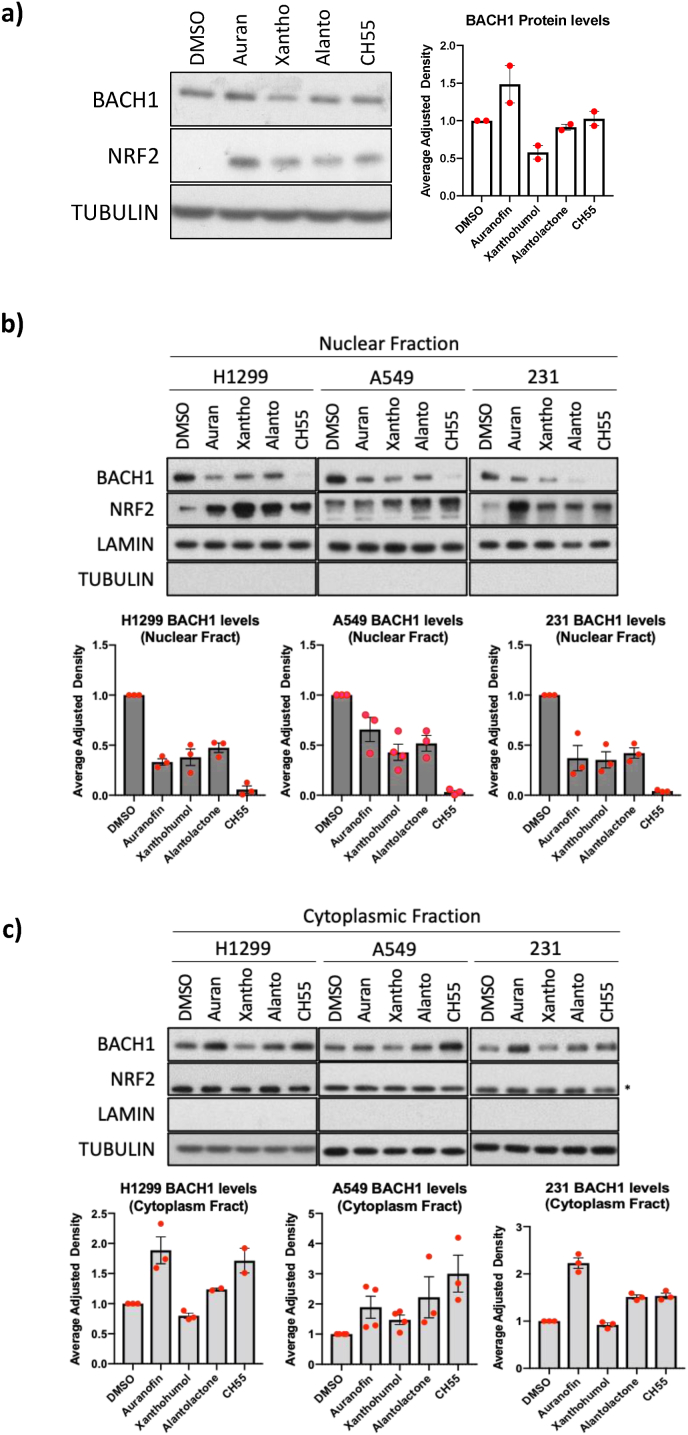
**Identified compounds reduce BACH1 nuclear levels. a)** H1299 cells were treated with vehicle (DMSO) or with auranofin (0.5 μM), xanthohumol (10 μM), alantolactone (3 μM) and CH55 (5 μM). After 6 h cells were lysed for whole cell lysate and the levels of the indicated proteins were analysed. Left figure shows a representative Western blot, and right figures shows the quantification of BACH1 protein levels normalised to TUBULIN levels. Data (n = 2) represent means ± SD and are expressed relative to the DMSO-treated samples. **b and c)** Lung cancer cells (H1299 and A549), or breast cancer cells (MDA-MB-231) cells were treated with vehicle (DMSO) or with auranofin (0.5 μM), xanthohumol (10 μM), alantolactone (3 μM) and CH55 (5 μM). After 6 h cells were lysed and fractionated in nuclear (b) and cytoplasmic fractions (c) and the levels of the indicated proteins were analysed. Each figure shows a representative Western blot together with the quantification of BACH1 protein levels normalised to LAMIN levels (b) or TUBULIN levels (c); data (n = 3) represent means ± SD and are expressed relative to the DMSO-treated samples.

Once we confirmed the compounds activated the BACH1 signature (and a well-validated NRF2 target gene), we tested their effect on BACH1 and NRF2 protein levels, to ensure target engagement and to gain insights into their potential mechanism of action. Of the four compounds, only xanthohumol reduced total BACH1 protein levels (Fig. 4a), suggesting that the other three compounds do not affect BACH1 protein stability. To date, all known BACH1 functional inhibitors act by reducing BACH1 nuclear levels, either by inducing its degradation or its nuclear exclusion [[37], [38], [39], [40], [41],^50^]. We therefore tested the compounds effect on nuclear and cytoplasmic BACH1 levels. All four compounds reduced BACH1 nuclear levels in the three cancer cell lines tested (Fig. 4b and c) and increased nuclear NRF2 levels (Fig. 4a and b and Suppl. Fig. S4a), further supporting their dual activity as functional BACH1 inhibitors and NRF2 activators. This dual BACH1/NRF2 targeting is not unexpected as the four compounds have electrophilic moieties (Fig. 3a). While electrophilicity alone is insufficient to inhibit BACH1, as we have shown by the lack of BACH1 inhibition by potent electrophiles such as SFN or CDDO [[37], [38], [39]], some electrophiles do inhibit BACH1 [37,38], likely due to the high abundance of potentially reactive cysteines within BACH1.

To further characterise the activity of these compounds as BACH1 functional inhibitors, we investigated their mechanism of action in more detail. Auranofin, alantolactone and CH55 reduced BACH1 nuclear levels while inducing its cytoplasmic accumulation (Fig. 4b and c), suggesting a nuclear exclusion or cytoplasmic sequestering mechanism. Although BACH1 can be exported from the nucleus via nuclear export receptor XPO1/CRM1-dependent [50] and -independent mechanisms [38], inhibition of CRM1 using KPT-330, a potent and selective CRM1 inhibitor, did not impair the compounds effects (Suppl. Fig. S4b), suggesting a CRM1-independent mechanism, in agreement with previous results for other electrophilic compounds [38]. Therefore, the precise mechanisms of action and the molecular players involved remain to be elucidated.

**Fig. 5:**
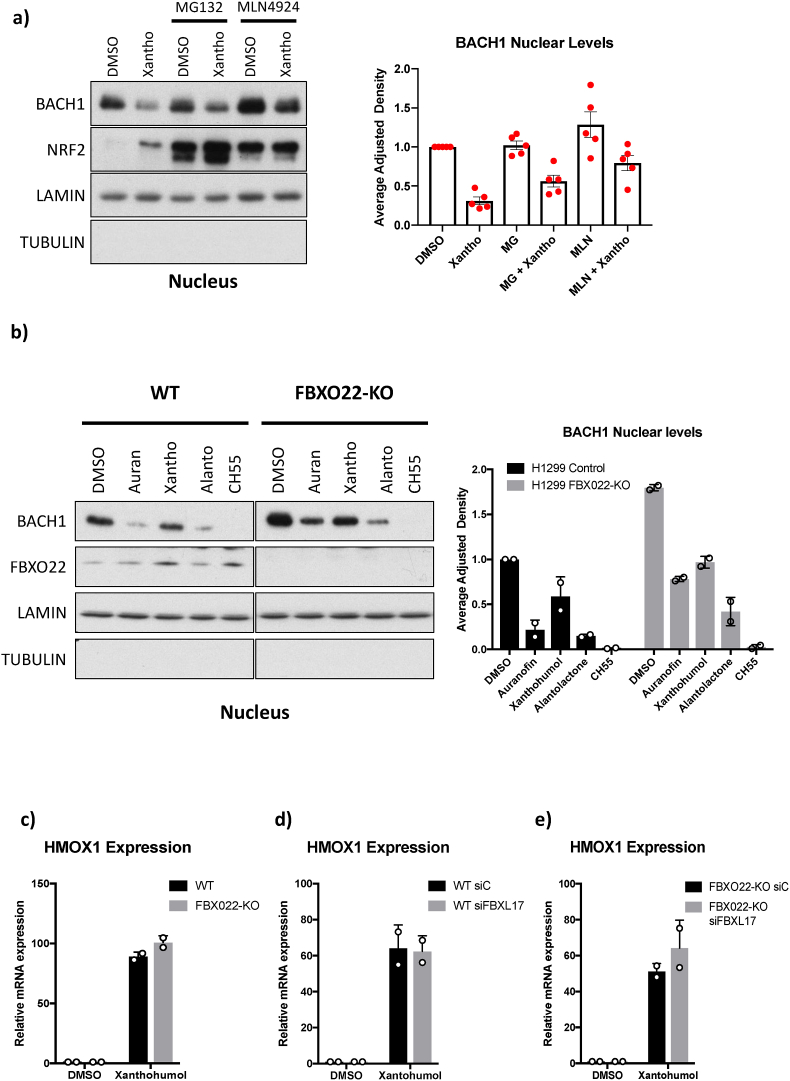
**Mechanism of action of xanthohumol. a)** H1299 cells were treated with either DMSO, 20 μM MG-132 (MG) or 2 μM MLN-4924 (MLN). Two hours later the cells were treated with either DMSO or xanthohumol (10 μM) and 6 h later cells were lysed and fractionated. The levels of the indicated nuclear proteins were analysed by Western blot (left panel). Right panel shows the quantification of BACH1 protein levels normalised to LAMIN levels; data (n = 4-5) represent means ± SD and are expressed relative to the DMSO-treated samples. **b)** H1299 control (WT) or FBXO22-KO cells were treated with either DMSO or the identified compounds as indicated. Five hours later cells were fractionated and the levels of the indicated nuclear proteins were analysed by Western blot (left panel). Right panel shows the quantification of BACH1 protein levels normalised to LAMIN levels; data (n = 2) represent means ± SD and are expressed relative to the control DMSO-treated samples. **c)** H1299 control (WT) and FBXO22-KO cells were treated with either DMSO or xanthohumol (10 μM). 16 h later, cells were lysed, and mRNA levels of *HMOX1* were analysed by real-time qPCR. Data (n = 2) represent means ± SD and are expressed relative to the control DMSO-treated sample. **d and e)** H1299 WT cells (d) or FBXO22-KO cells (e) were transfected with either siControl or siFBXL17. 36 h later, cells were treated with either DMSO or xanthohumol (10 μM) for 16 h. After that, cells were lysed, and mRNA levels of *HMOX1* were analysed by real-time qPCR. Data (n = 2) represent means ± SD and are expressed relative to the control DMSO-treated sample.

Of the four compounds, xanthohumol was the only one that could potentially act via BACH1 degradation (Fig. 4a–c). While BACH1 levels can be regulated via several E3 ligases [41,51,52], degradation mediated by FBXO22 and FBXL17 (two E3 ligases that are part of Cullin-RING ligase complexes) is the best characterised. To address whether the reduction in BACH1 nuclear levels in response to xanthohumol involved Cullin E3-mediated proteasomal degradation, we used the proteasome inhibitor MG-132 and the neddylation inhibitor MLN-4924 that inactivates Cullin-RING E3 ubiquitin Ligases. Fig. 5a shows that neither MG132 nor MLN-4924 fully recovered BACH1 levels, although MLN-4924 might partially reduce the effect of xanthohumol. As a control, we used TBE56, a validated BACH1 degrader [37] whose effect was completely abolished by both MG-132 and MLN-4924 (Suppl. Fig. S5a). These results suggest that the effect of xanthohumol in reducing nuclear BACH1 might in part be mediated via Cullin E3-ligase-mediated proteasomal degradation. However, experiments in cells with and without FBXO22 and/or FBXL17 showed that FBXO22 is not necessary for the xanthohumol-mediated reduction in BACH1 nuclear levels (Fig. 5b) and neither of these E3 ligases is needed for the subsequent *HMOX1* induction (Fig. 5c–e). We also included the three other identified compounds, and as expected based on their mechanism of action, FBXO22 deficiency did not affect their ability to reduce BACH1 nuclear levels (Fig. 5b).

Given that the effect of xanthohumol on BACH1 was not reversed by inhibition of the ubiquitin-proteasome system, we next explored whether an alternative degradation pathway could be involved. Autophagy is an established mechanism for the turnover of several transcription factors, including BACH1 [53], and thus, we investigated whether this pathway might contribute to the reduction in BACH1 levels observed upon xanthohumol treatment. To assess this, we used two inhibitors: the antimalarial drug chloroquine which blocks autophagy by inhibiting autophagosome and lysosome fusion, and bafilomycin A1, an antibiotic that inhibits autophagic flux by preventing endosomal and lysosomal acidification. Inhibition of autophagy did not restore nuclear BACH1 levels in response to xanthohumol, suggesting that autophagy pathways are not involved (Suppl. Fig. S5b). Although we used multiple strategies, the precise mechanism of action and associated pathway(s) could not be defined, underscoring the need for further studies.

**Fig. 6:**
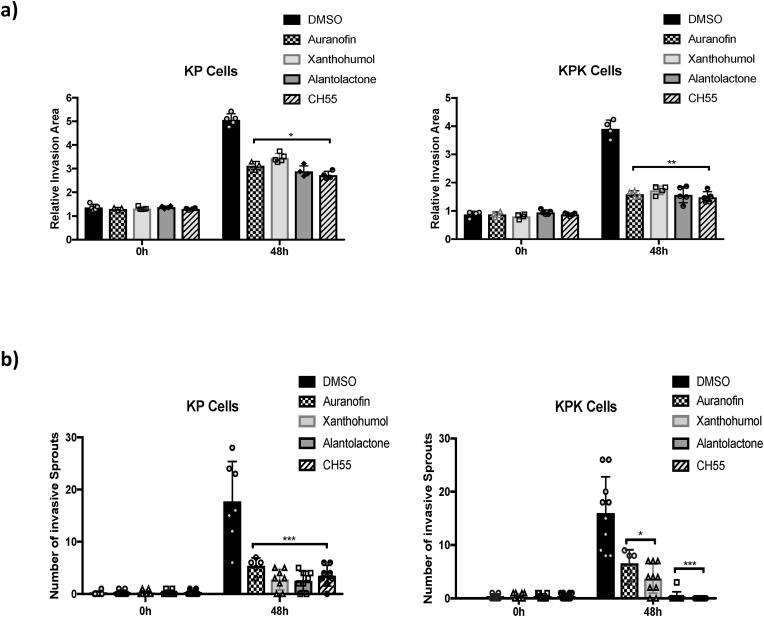
**Identified BACH1-targeting compounds reduce lung cancer cell invasion. a)** KP and KPK spheroids were embedded in collagen I and treated with DMSO (vehicle), auranofin (0.5 μM), xanthohumol (10 μM), alantolactone (3 μM), or CH55 (5 μM). 24 h later media with the compound was exchanged with fresh media plus compound. Spheroid invasion was monitored using the Incucyte Live-Cell Imaging System, and invasion area was quantified at 0 h and 48 h. DMSO-treated spheroids showed increased invasion over time, whereas spheroids exposed to the compounds displayed reduced invasion areas at 48 h. Data represent mean ± SD (n = 3-5); statistical significance was determined by one-way ANOVA (∗p < 0.05, ∗∗p < 0.01). **b)** The number of invasive sprouts formed by KP and KPK spheroids was quantified at 0 h and 48 h using bright-field microscopy. At 48 h, DMSO-treated spheroids formed numerous invasive projections, while compound-treated spheroids showed fewer sprouts. Data represent mean ± SD (n = 5-10); statistical significance was determined by one-way ANOVA (∗p < 0.05, ∗∗∗p < 0.001).

*Functional studies:* One of the best-characterised functions of BACH1 is its role in promoting tumour metastasis. Consistent with this, both genetic depletion and pharmacological inhibition of BACH1 reduce cancer cell migration and invasion [[3], [4], [5],^37^,^38^,^54^]. Importantly, these effects are BACH1-specific, as NRF2 activation alone does not impair cancer cell migration or invasion [37,38]. As a result, cell migration assays have been widely used as a functional readout for selectively assessing BACH1 inhibition. Based on this, we hypothesised that the four compounds identified in our screen would reduce cancer cell migration and invasion. To test this, we evaluated their impact on the migratory capacity of a panel of human (H1299, A549, MDA-MB-231) and murine (KP and KPK) cancer cell lines. Despite all compounds being confirmed as BACH1 functional inhibitors (demonstrated by reduced nuclear BACH1 levels and induction of validated BACH1 target genes) only xanthohumol consistently inhibited migration across all five cell lines. Alantolactone reduced migration in three cell lines, and CH55 and auranofin were effective only in H1299 cells (Suppl. Fig. S6a). This finding was unexpected, as migration assays have been widely used as a reliable proxy for BACH1 inhibition, including in our own previous studies. We therefore considered several possible explanations for these findings: (a) as these compounds are pleiotropic, they may modulate pathways that counteract the anti-migratory effects associated with BACH1 inhibition; (b) differences in mechanism of action, potency, or kinetic profiles/timing of the inhibitors may influence the results; and (c) conventional migration assays could produce false negatives as they do not involve matrix remodelling, a key process regulated by BACH1 that is not captured in migration settings. To address the latter, we used a more physiologically relevant 3D lung cancer spheroid invasion model. In this assay, cancer cells are first seeded in ultra-low attachment plates to allow the formation of 3D spheroids, which are subsequently embedded in a collagen matrix, allowing assessment of invasion into the surrounding matrix. The effect of the compounds on the spheroid invasion areas were quantified (Fig. 6a). In this model, all compounds significantly reduced invasion in the two lung cancer cell lines. Notably, this effect was observed in KP cells, which express an active KEAP1, as well as in KPK cells, which lack KEAP1 [44], implying that the anti-invasive effect is not mediated through the KEAP1/NRF2 pathway. A complementary assay measuring the number of invasive sprouts per spheroid produced similar results (Fig. 6b), although with greater variability, further supporting the anti-invasive activity of the four compounds. Together, these findings indicate that conventional migration assays may underestimate the functional impact of certain BACH1 inhibitors, whereas 3D invasion models more accurately capture their anti-metastatic potential.

Overall, these results demonstrate that the compounds identified in our screen function as BACH1 functional inhibitors and exhibit anti-invasive activity.

## 4 Discussion

The BACH1/NRF2 axis has emerged as therapeutically relevant across a wide range of conditions. NRF2 activation is well established as beneficial in conditions associated with oxidative stress and inflammation (reviewed in Ref. [55]). Independently, pharmacological inhibition or genetic depletion of BACH1 has also demonstrated therapeutic value in similar contexts, particularly in conditions driven by or linked to inflammation and oxidative stress [2,17,[22], [23], [24], [25],^27^,^28^]. Importantly, BACH1 inhibition has additional relevance in cancer, where it reduces tumour cell invasion and metastatic potential [[3], [4], [5], [6], [7],^54^], highlighting BACH1 as an attractive therapeutic target across multiple disease settings.

Although both NRF2 activation and BACH1 inhibition enhance cytoprotective gene expression, their outcomes are not identical either in magnitude or duration. For example, BACH1 inhibition induces a stronger and more sustained *HMOX1* induction than NRF2 activation alone [39,56] a difference also highlighted in the present study. These distinctions suggest that BACH1 inhibition engages regulatory mechanisms that extend beyond simple derepression of NRF2 target genes, with potential consequences for therapeutic efficacy.

In conditions marked by chronic inflammation and/or oxidative stress, these differences raise the possibility that combined BACH1 inhibition and NRF2 activation could provide a more robust and sustained anti-inflammatory and antioxidant response, potentially achieving greater therapeutic efficacy than inhibiting each pathway individually. In contrast, current evidence offers no clear rationale for favouring compounds with dual BACH1/KEAP1 inhibition over selective BACH1 inhibition in the context of cancer therapy. However, given that many tumours display hyperactive NRF2 signalling, the use of a dual BACH1/KEAP1 inhibitor versus the use of a selective BACH1 inhibitor might not be significantly different. This possibility needs to be further studied considering the systemic effects and the impact on tumour microenvironment of such compounds.

Despite the growing interest in BACH1 as a therapeutic target, relatively few validated BACH1 inhibitors have been reported to date [21,24,29,[37], [38], [39], [40], [41], [42]], especially when compared with the abundance of well-characterised NRF2 activators. In this study, we describe the optimisation and validation of a robust cellular screening assay designed to identify novel functional BACH1 inhibitors. Using a small-compounds library, we performed a proof-of-concept screen and implemented a stepwise validation pipeline. All hits identified after this validation process were confirmed as functional BACH1 inhibitors with anti-invasive activity across multiple cell lines, demonstrating the robustness and suitability of this assay as a screening platform.

Previous work from our group identified CDDO-derivatives, including CDDO-Me (Bardoxolone) and CDDO-DFPA (Omaveloxolone), as potent BACH1/KEAP1 inhibitors [38]. However, Bardoxolone has been associated with dose-limiting toxicities, including cardiovascular adverse effects [57], which restrict its therapeutic window and long-term applicability *in vivo*. These safety concerns highlight the need for novel BACH1/KEAP1-targeting compounds that retain potent inhibitory activity while exhibiting improved tolerability.

Notably, the compounds identified in the present study include both approved drugs and well-characterised natural products with established anti-inflammatory and antioxidant properties. Auranofin is an FDA-approved gold salt used in rheumatoid arthritis whose primary mechanism of action is inhibition of thioredoxin reductases (TrxRs) [58,59]. Xanthohumol is a natural compound, prenylated chalcone, that belongs to the flavonoid family, with reported antimicrobial, anti-inflammatory, and antioxidant activities [60,61] and demonstrated safety in phase I and phase II trials [62,63]. Alantolactone is a natural compound, member of the sesquiterpene lactone class with anti-inflammatory and antioxidant effects [64,65] and has been tested in several animal models without reported toxicity [66,67]. CH55 is a synthetic retinoid with high affinity for RAR-α and RAR-β and antifibrotic activity [[68], [69], [70]] and has not been yet tested *in vivo*. Together, these properties support the translational relevance of the chemical scaffolds identified here.

The dual activity of these compounds as both BACH1 and KEAP1 functional inhibitors may be explained by their potential to covalently modify reactive cysteine residues present in these proteins. Both KEAP1 and BACH1 contain multiple cysteines that are known to function as redox sensors or sites of electrophilic modification. In the case of KEAP1, known reactive cysteines include Cys151, Cys273, Cys288 [71], and mass spectrometry analysis of recombinant KEAP1 have shown that xanthohumol alkylates at least 2−3 cysteine sulfhydryl groups, including Cys151 [[72], [73], [74]]. Similarly, BACH1 contains several cysteine residues, such as Cys107, Cys122, Cys557 and Cys574, which are sensitive to electrophiles, oxidants and/or *S*-nitrosylation [51,75,76], and modification of these cysteines plays a role in BACH1 regulation [52]. Therefore, it is not surprising that some electrophiles may simultaneously inhibit KEAP1 and BACH1, accounting for the dual effects observed in our screen. However, electrophilicity alone is not sufficient for BACH1 inhibition, as potent electrophilic NRF2 activators like sulforaphane, CDDO, or TBE-31 do not affect BACH1 [37,38,42]. This indicates that while cysteine reactivity likely contributes to BACH1 inhibition, additional structural or biochemical determinants are required. Our findings do not provide evidence for direct binding of the compounds to BACH1, nor do they elucidate their precise mechanism(s) of action. Comprehensive characterization of these mechanisms, including potential direct interactions with BACH1 and identification of critical residues, will require further investigation using biophysical and structural approaches such as Surface Plasmon Resonance (SPR) and CP motif analysis, and represents an important direction for future research.

Although the primary goal of this study was to identify BACH1 functional inhibitors, we did not include steps to explicitly exclude dual BACH1/KEAP1 inhibitors, given the already established potential therapeutic value of such compounds. Interestingly, all hits identified in this proof-of-concept screen displayed dual BACH1 inhibitory and NRF2 activating properties, suggesting that compounds with dual activity may be more common than previously appreciated. Importantly, our data (Suppl. Fig. S3a and S3b) demonstrate that HMOX1 induction by these hits is BACH1-dependent and NRF2-independent. Nevertheless, despite BACH1 being the dominant regulator of *HMOX1* and the screening threshold being set to capture strong effects (likely BACH1-dependent), we cannot fully exclude the possibility that some hits may activate *HMOX1* through BACH1-independent mechanisms. More broadly, any reporter-based primary screen centred on a single target gene cannot completely rule out effects mediated by alternative pathways. To address this, we incorporated multiple selection criteria and validation steps to confirm functional BACH1 inhibition by identified hits, including stringent thresholds to exclude weak *HMOX1* inducers (such as NRF2 activators) and secondary validation using notably a BACH1-specific transcriptional signature. In the future, specificity could be further enhanced by implementing parallel high-throughput assays in the screening pipeline, such as using HMOX1-luciferase in BACH1-knockout cells to exclude BACH1-independent HMOX1 activation, or ARE-luciferase reporter cells to filter out dual BACH1/KEAP1 functional inhibitors. Together, these modifications would further improve pathway specificity while preserving the core strength of the platform: identification of functionally active BACH1 inhibitors in a cellular context.

Additionally, our findings demonstrate that conventional migration assays may fail to fully capture the anti-invasive effects of BACH1 inhibitors, leading to false negative results and an underestimation of their therapeutic potential. Given the critical role of BACH1 in promoting invasion rather than simply migration, our findings suggest that 3D invasion assays better recapitulate the biological processes modulated by BACH1 and should be prioritised in future drug screening pipelines.

In summary, this work establishes a robust screening platform for the identification of functional BACH1 inhibitors and provides new chemical scaffolds with potential for future therapeutic development.

## Funding

This work was supported by 10.13039/501100000289Cancer Research UK (C52419/A22869 to LV, DW and MH), Ninewells Cancer Campaign (DKS), the 10.13039/501100000265Medical Research Council (MR/W023806/1 to 10.13039/100013593ADK) the 10.13039/501100004359Swedish Research Council (2018-02318 and 2022-00971 to 10.13039/100019100VIS, 2021-03138 to CW), the Swedish Cancer Society (23-3062 to 10.13039/100019100VIS, 22-0612FE to CW), Assar Gabrielsson Research Foundation (to KXA, AAHP, DR, CW, and 10.13039/100019100VIS), the 10.13039/501100003748Swedish Society for Medical Research (2018; S18-034 to 10.13039/100019100VIS), the 10.13039/501100004063Knut and Alice Wallenberg Foundation, and the 10.13039/501100017018Wallenberg Centre for Molecular and Translational Medicine (to 10.13039/100019100VIS). For the purpose of open access, the authors have applied a Creative Commons Attribution (CC BY) licence to any Author Accepted Manuscript version arising from this submission.

## CRediT authorship contribution statement

**Kevin X. Ali:** Investigation, Visualization, Writing – review & editing. **Donika Klenja-Skudrinja:** Formal analysis, Investigation, Visualization, Writing – review & editing. **Maureen Higgins:** Investigation. **David Walker:** Investigation. **Yumna Sharaf:** Investigation. **Martin Dankis:** Formal analysis, Visualization. **Angana A.H. Patel:** Formal analysis, Visualization. **Dorota Raj:** Formal analysis, Visualization. **Jozefina J. Dzanan:** Visualization. **Esben B. Svenningsen:** Data curation, Methodology, Visualization. **Alistair Langlands:** Data curation, Methodology. **Thomas Poulsen:** Data curation, Methodology. **Tadashi Honda:** Resources. **Albena T. Dinkova-Kostova:** Conceptualization, Visualization, Writing – review & editing. **Clotilde Wiel:** Conceptualization, Supervision, Writing – review & editing. **Volkan I. Sayin:** Conceptualization, Supervision. **Laureano de la Vega:** Conceptualization, Data curation, Funding acquisition, Project administration, Supervision, Writing – original draft, Writing – review & editing.

## Declaration of competing interest

None.

## Data Availability

No data was used for the research described in the article.

## Appendix A Supplementary data

Supplementary Figure S1. **a)** *Related to* Fig. 1b*.* 10% of the sample was used to measure mRNA levels of BACH1 and KEAP1 to test for silencing efficiency.

Supplementary Figure S2. **Optimisation, quality control for the HMOX1-luciferase assay in 384-well plates and screen validation**. a) Effect of cell density on assay performance. Cells were plated into a 384 well plate at a range of densities from 4.6x10^4^ cells/cm^2^ (5000 cells/well) to 1.8x10^5^ cells/cm^2^ (20,000 cells/well), and were non-treated (UT), treated with DMSO, 5 μM or 10 μM hemin. There were 8 wells for each treatment at each cell density. The boxplot (left panel) displays the range of values from each treatment. The middle panel shows the Signal:Background (SB) and the right panel the rZ’ (both calculated using 10 μM hemin as the positive control, DMSO as the negative control). **b)** Effect of cell density on hit-picking ability in a blind spike-in assay. Three of the best performing cell densities were further examined in a blind spike-in assay, where random test wells were treated with either 10 μM hemin or 100 nM CDDO (left panel). Test wells with equivalent luminescence values to hemin-treated controls were all found to have been treated with hemin, and those with equivalent luminescence values to CDDO-treated controls were all found to have been treated with CDDO. Test wells with equivalent luminescence values to DMSO-treated controls were all found to be un-treated. Assay performance was measured using control well values, with SB ∼10 (middle panel), and rZ’ >0.5 for all tested densities. **c)** DMSO tolerance test. Assay performance was tested at a range of DMSO concentrations from 0 to 0.5% (the highest likely concentration required in a dose response experiment). The box plot (left panel) displays the range of luminescence values measured at the different DMSO concentrations, and after the different control treatments. SB (middle panel) is stable at ∼10 at all tested concentrations, however rZ’ (right panel) decreases at the highest DMSO concentrations. **d)** Table showing assay metrics per cell density: Pos_mean, mean of positive control (hemin)-treated wells; Pos_sd, standard deviation of hemin-treated wells; Neg_mean, mean of negative control (DMSO)-treated wells; Neg_sd, standard deviation of DMSO-treated wells; P_CV, coefficient of variation (SD/Mean ∗ 100) in hemin-treated wells; N_CV, coefficient of variation in DMSO-treated wells; SB, signal: background between hemin- and DMSO-treated wells (Pos_Mean/Neg-Mean); rSB, SB calculated using median instead of mean; Z′, assay QC metric calculated using equation 1(3∗(*SD*(h*emin*) +*SD*(*DMSO*))*mean*(h*emin*)−*mean* (*DMSO*)); rZ′, assay QC metric calculated using Median and MAD in place of mean and SD. **e)** Distribution of blank-corrected Luminescence values across the 4480 Test wells (purple), and control wells (DMSO, 224 wells, red; hemin, 224 wells, green; CDDO, 224 wells, blue) in all assay plates – graph is cropped at Luminescence = 6x10^5^ for clarity, this excludes some of the test wells with higher Luminescence values. **f)** Fold change (over DMSO control wells, normalised per-plate) in Test wells and control wells. DMSO-treated wells are distributed around 1; CDDO-treated wells around 2.5; and hemin-treated wells around 10. Most Test wells are distributed around 1, with some exhibiting increased Luminescence (up to >100 fold change over DMSO), 70 Test wells had a fold change >2.5 over DMSO. **g)** Summary of Assay QC measures: rZ’ was >0.5 in all 14 assay plates (upper panel), and SB > 8.7 in all assay plates (lower panel). **h)** Comparison of fold change value for each compound in each replicate (left panel). The average fold change value for each compound plotted against the difference in values between replicate 1 and replicate 2 (fold change^Rep1^ – fold change^Rep2^), (right panel). **i)** Table showing the 34 compounds identified in the primary screen including their average of fold-change over DMSO control between replicates (Av foldchange) and their difference in fold-change between replicates 1 and 2 (Diff foldchange). **j)** H1299 HMOX1-Luc cells were treated with a range of concentrations (0.01 – 30 μM) for the indicated compounds; 16 h later luciferase activity was measured. The box shows the concentration of the compound that produces a level of HMOX1 similar to the one obtained with hemin 10 μM (indicated by the red line). **k** and **l)** H1299 cells were treated with the indicated range of concentrations for the validated BACH1 inhibitors (k) or the indicated compounds (l); 48 h later cell viability was calculated relative to the DMSO-treated controls using Alamar Blue. The red point indicates either the concentration of the validated inhibitors normally used to inhibit BACH1 (k) or the concentration of the compound that leads to a 7.8-fold HMOX1-luc induction (l); the red line indicates the 70% survival point. Data (n = 3) represent means ± SD and are expressed relative to the DMSO-treated sample.

Supplementary Figure S3. **a)** H1299 HMOX1-Luc BACH1-KO cells were treated with either DMSO, auranofin (0.5 μM), xanthohumol (10 μM), alantolactone (3 μM) or CH55 (5 μM). 16 h later, luciferase levels were measured. Data (n = 2) represent means ± SD and are expressed relative to the DMSO treated sample. **b)** WT, BACH1-KO, NRF2-KO or double NRF2/BACH1-KO HaCaT cells were treated with either DMSO, auranofin (0.5 μM), xanthohumol (10 μM), alantolactone (3 μM) or CH55 (5 μM). 16 h later, cells were lysed, and mRNA levels of *HMOX1* were analysed by real-time qPCR. Data (n = 3) represent means ± SD and are expressed relative to the DMSO treated sample.

Supplementary Figure S4. **a)** *Related to* Fig. 4b*.* Quantification of NRF2 nuclear protein levels normalised to LAMIN protein levels; data (n = 3) represent means ± SD and are expressed relative to the DMSO-treated samples. **b)** H1299 cells were treated with either DMSO, or 1 μM of the CRM1 inhibitor KPT-330 (KPT). Two hours later the cells were treated with either DMSO, alantolactone (3 μM), CH55 (5 μM) or auranofin (0.5 μM) and 6 h later cells were lysed and fractionated in nuclear (top panel) and cytoplasmic (bottom panel) fractions. The levels of the indicated proteins were analysed by Western blot (left panels). Right panels show the quantification of BACH1 protein levels normalised to LAMIN (nuclear fraction) or TUBULIN (cytoplasmic fraction) levels; data (n = 2) represent means ± SD and are expressed relative to the DMSO-treated samples.

Supplementary Figure S5. **a)** H1299 cells were treated with either DMSO, 20 μM MG-132 (MG) or 2 μM MLN-4924 (MLN). Two hours later the cells were treated with either DMSO or TBE56 (100 nM) and 6 h later cells were lysed and fractionated. Graph shows the quantification of BACH1 nuclear protein levels normalised to LAMIN levels; data (n = 3-5) represent means ± SD and are expressed relative to the DMSO-treated samples. **b)** H1299 cells were treated with either DMSO, 120 μM Chloroquine (CQ) or 100 nM Bafilomycin 1A (BAF1A). Two hours later the cells were treated with either DMSO or xanthohumol (10 μM), and 6 h later cells were fractionated. The levels of the indicated nuclear proteins were analysed by Western blot (left panel). Right panel shows the quantification of BACH1 protein levels normalised to LAMIN levels; data (n = 3) represent means ± SD and are expressed relative to the DMSO-treated samples.

Supplementary Figure S6. **a)** A549, H1299, MDA-MB-231, KP, and KPK cells were treated with DMSO (0.1%, v/v), auranofin (0.5 μM), xanthohumol (10 μM), alantolactone (3 μM), or CH55 (5 μM) and assessed using transwell migration assays. Migration was quantified after 16 h and expressed relative to the DMSO-treated control. Each symbol represents an independent biological replicate (n = 3–6 per condition). Horizontal bars indicate mean values. Statistical significance was determined by one-way ANOVA (∗p < 0.05, ∗∗p < 0.01, ∗∗∗p < 0.001).
