# Supplementary material for: Development of a high-throughput screening platform for identification of functional BACH1 inhibitors reveals compounds with anti-invasive potential
Source: Redox Biol. 2026 Apr 24;94:104187. doi: 10.1016/j.redox.2026.104187 (PMC13158619; doi:10.1016/j.redox.2026.104187)
Supplement: Multimedia component 1 [file mmc1.pptx]

## Slide 1
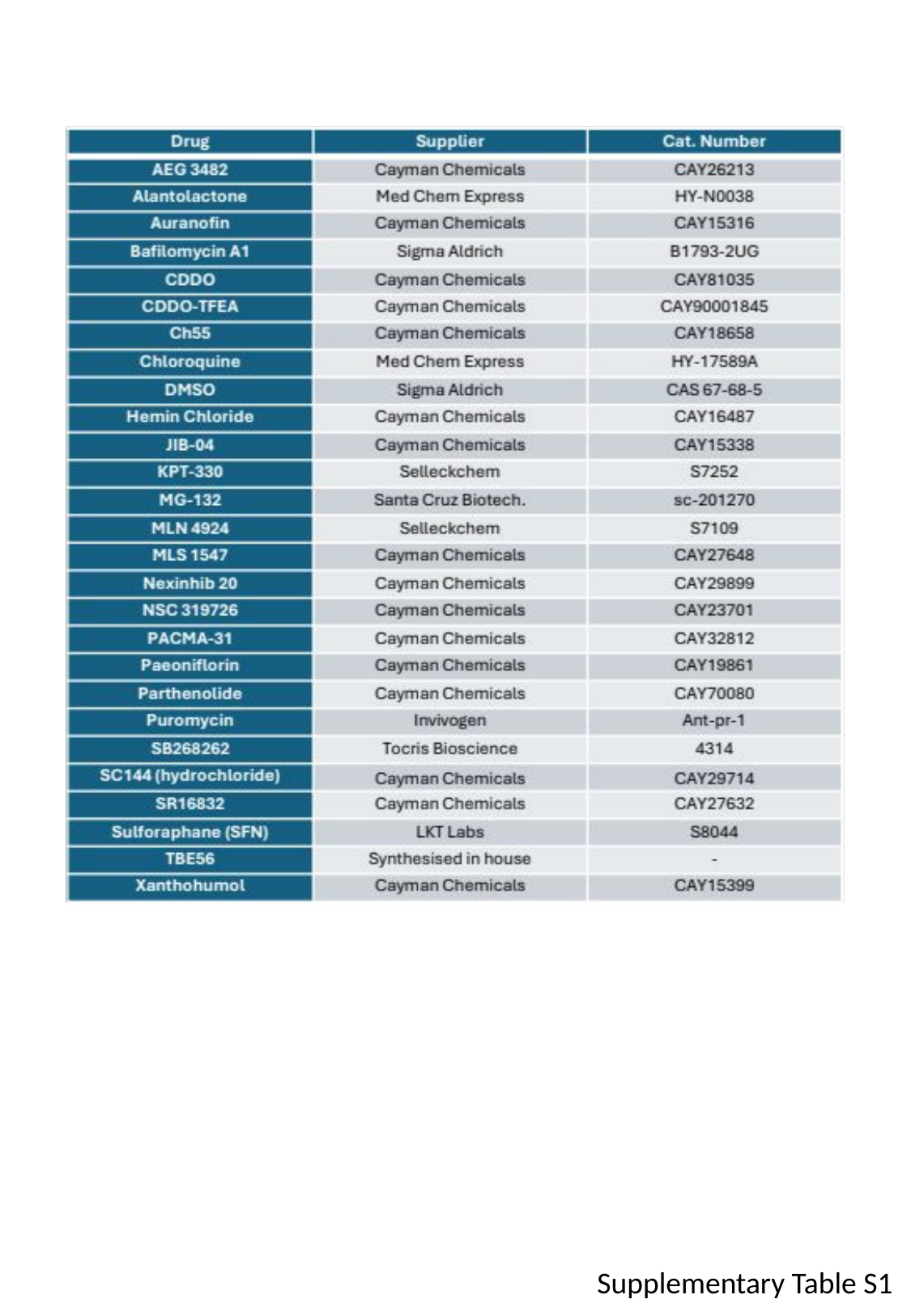

Supplementary Table S1

## Slide 2
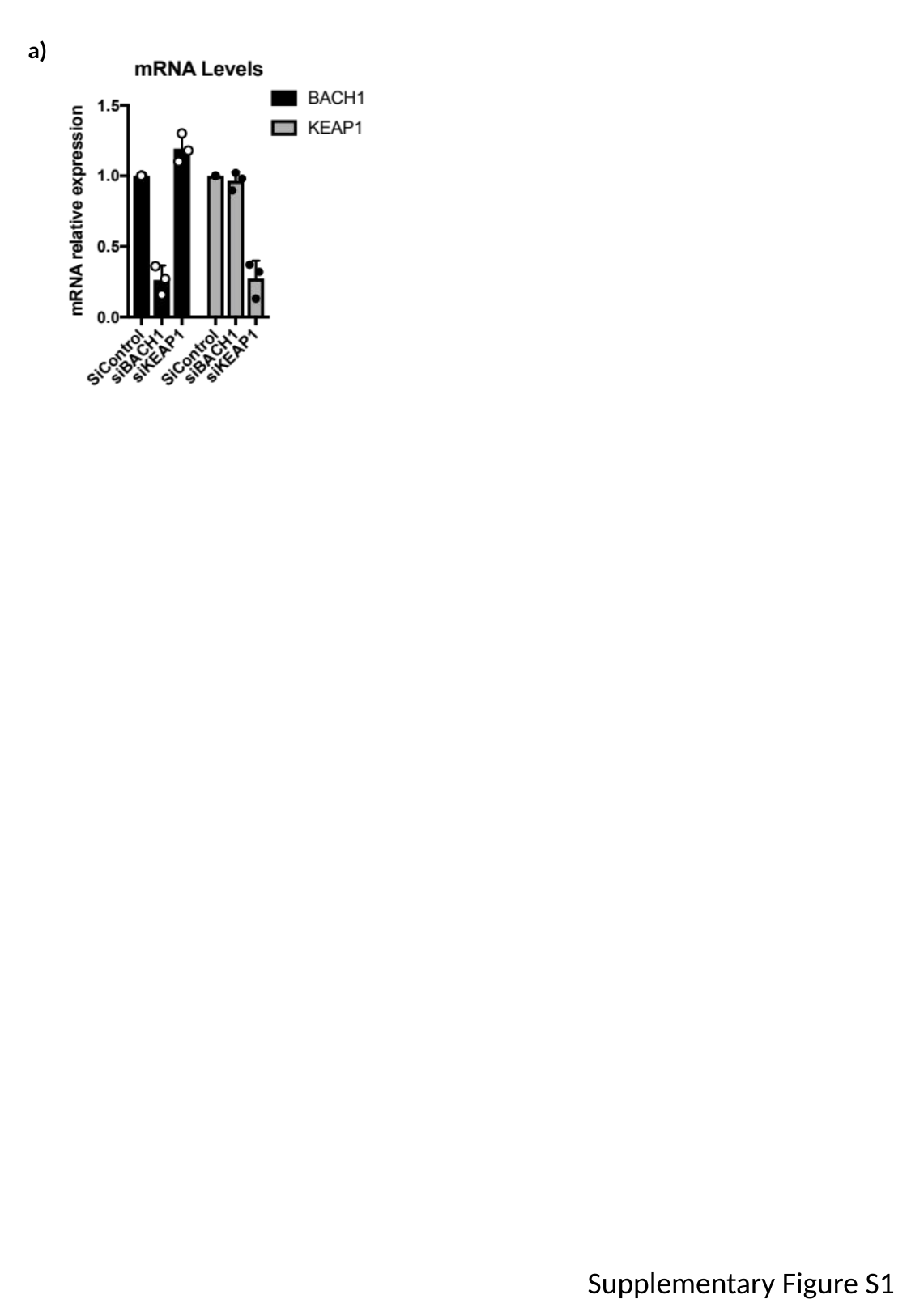

a)
Supplementary Figure S1

## Slide 3
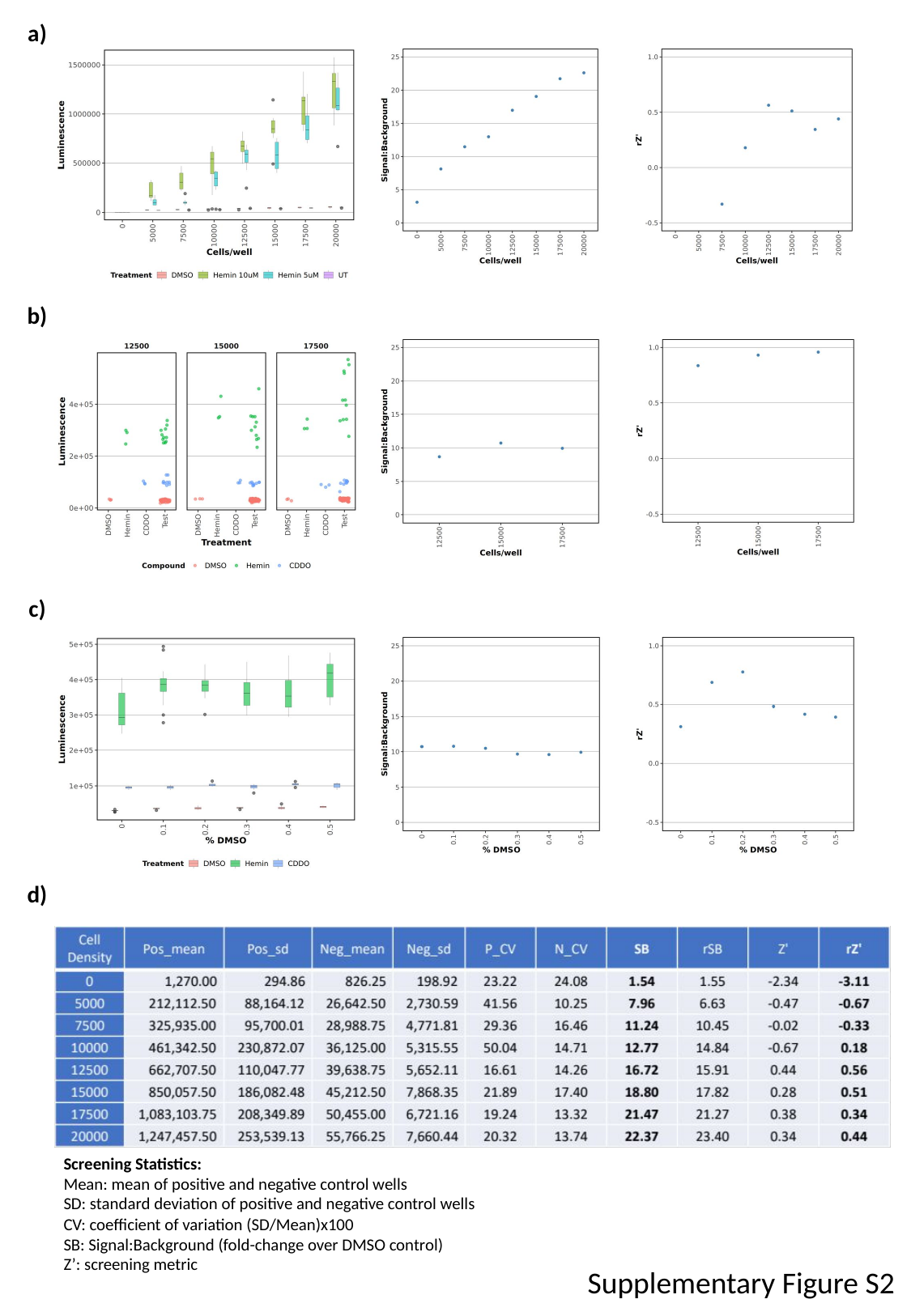

a)
b)
c)
d)
Supplementary Figure S2

## Slide 4
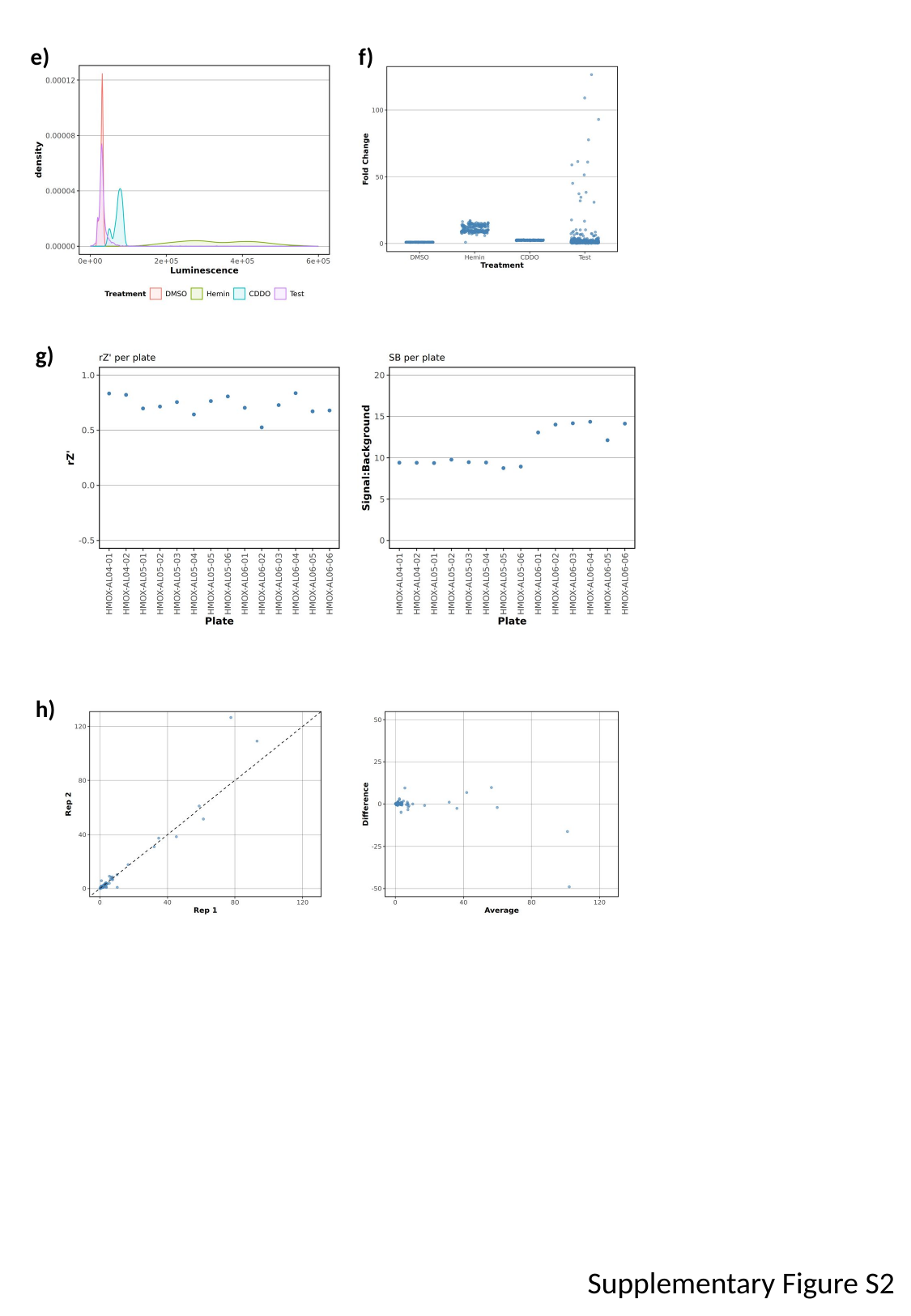

e)
f)
g)
h)
Supplementary Figure S2

## Slide 5
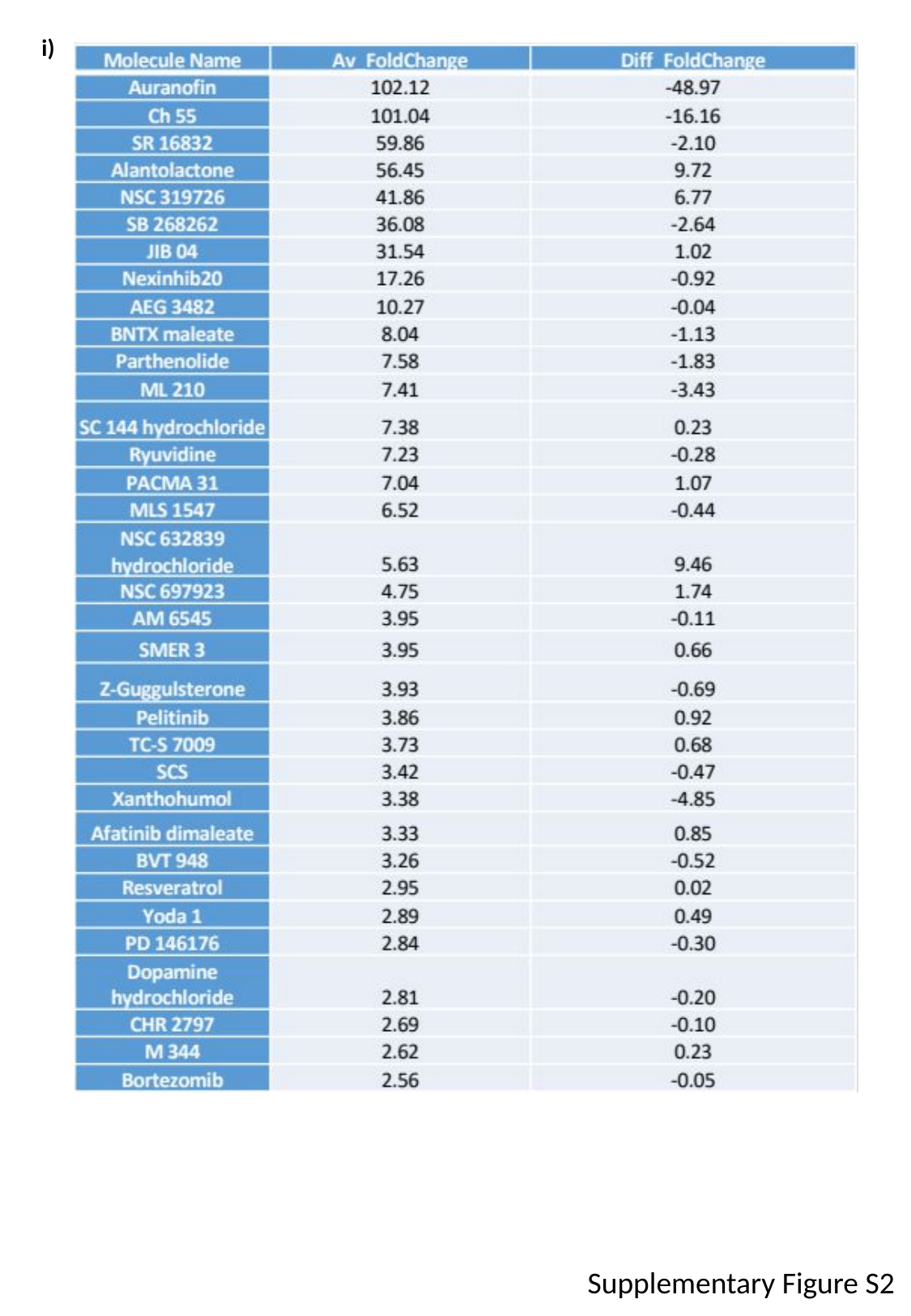

i)
Supplementary Figure S2

## Slide 6
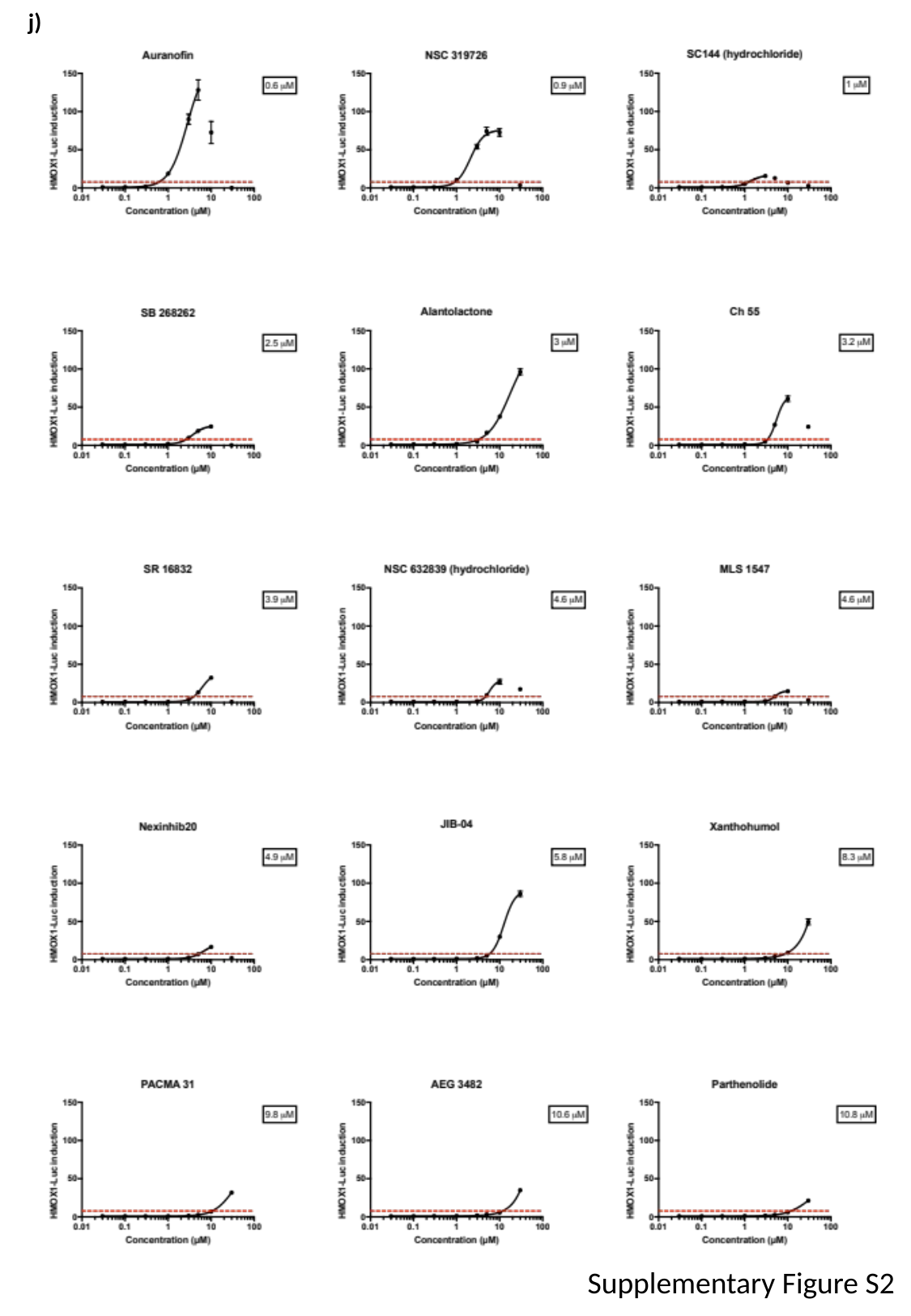

j)
Supplementary Figure S2

## Slide 7
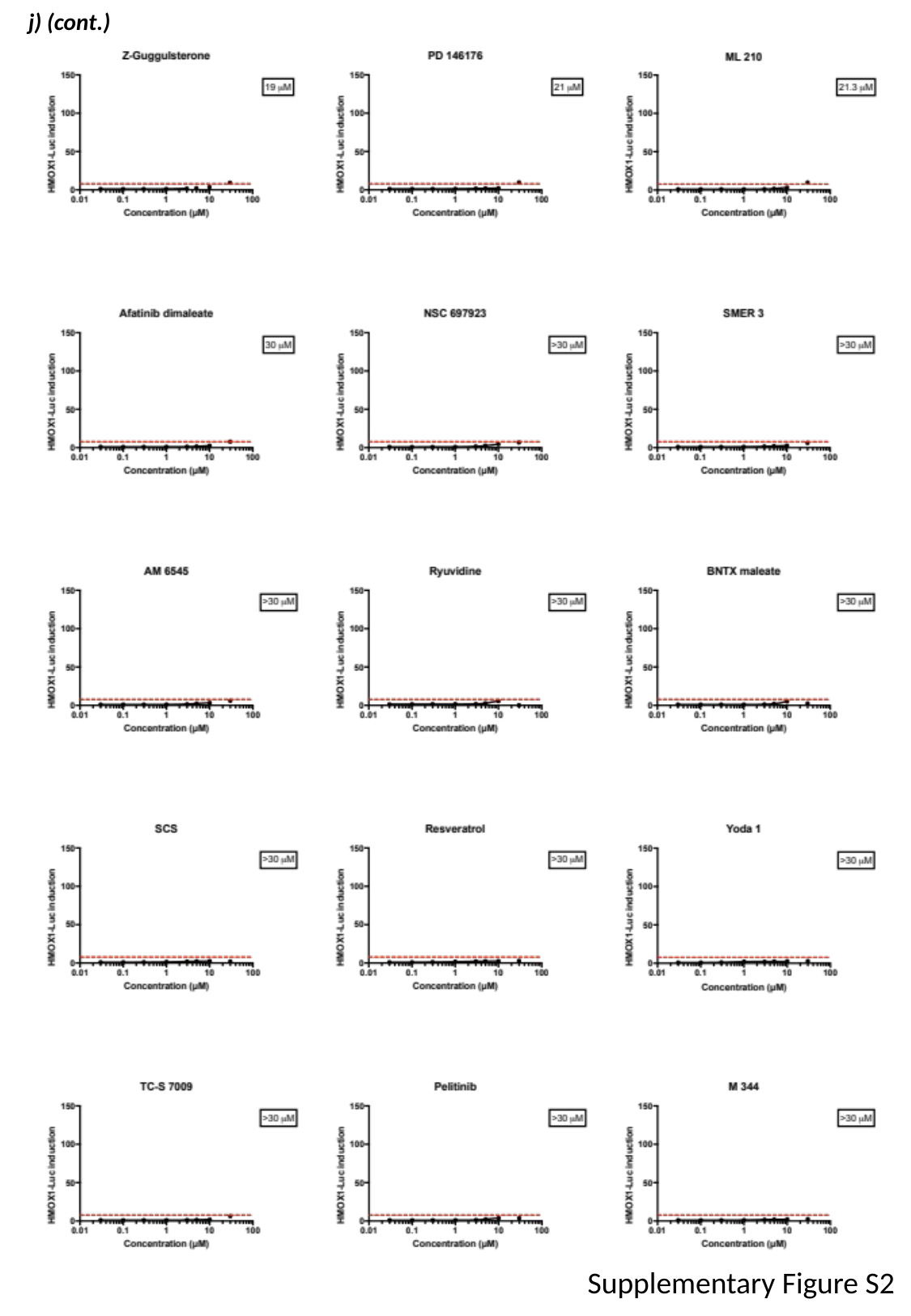

j) (cont.)
Supplementary Figure S2

## Slide 8
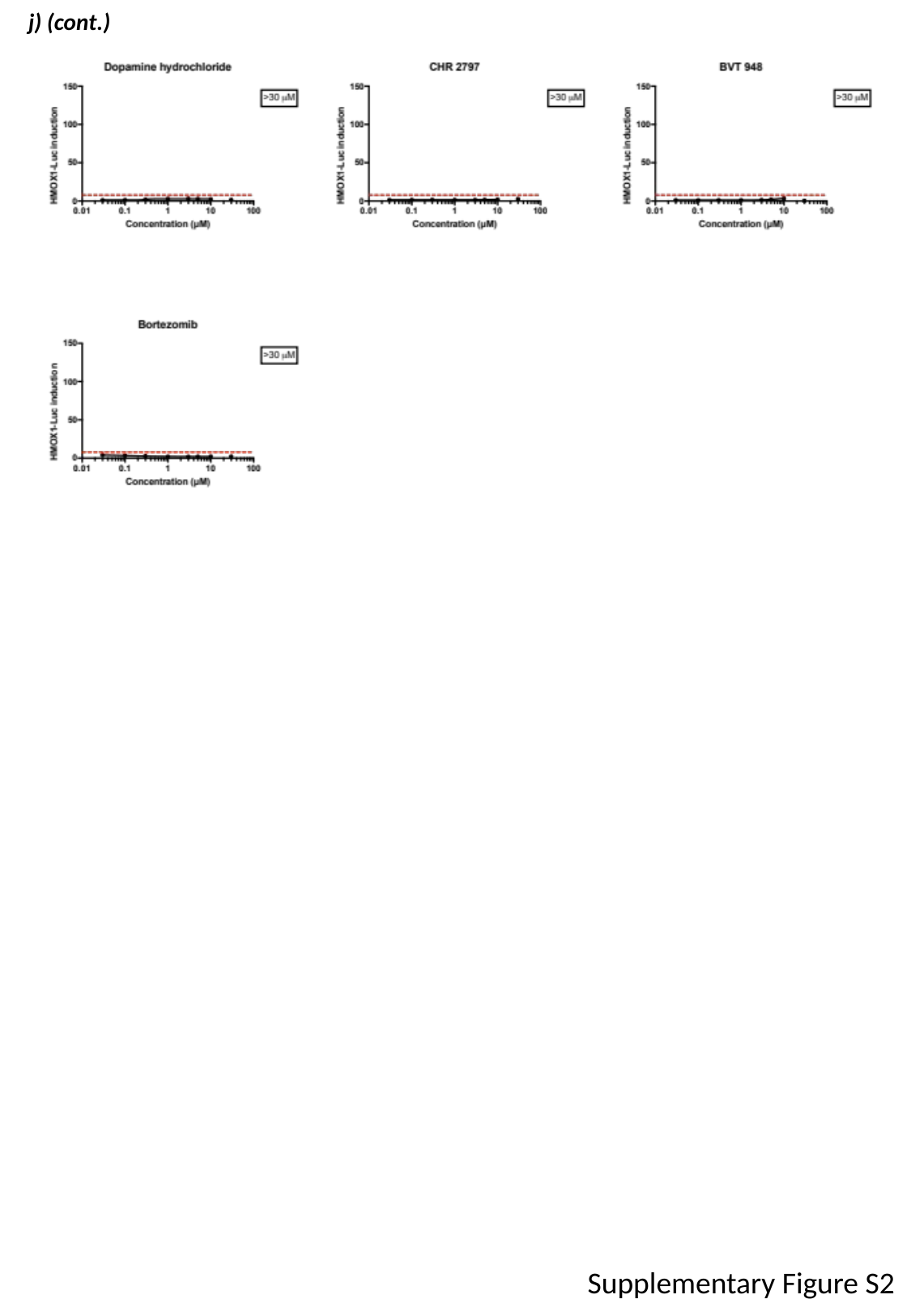

j) (cont.)
Supplementary Figure S2

## Slide 9
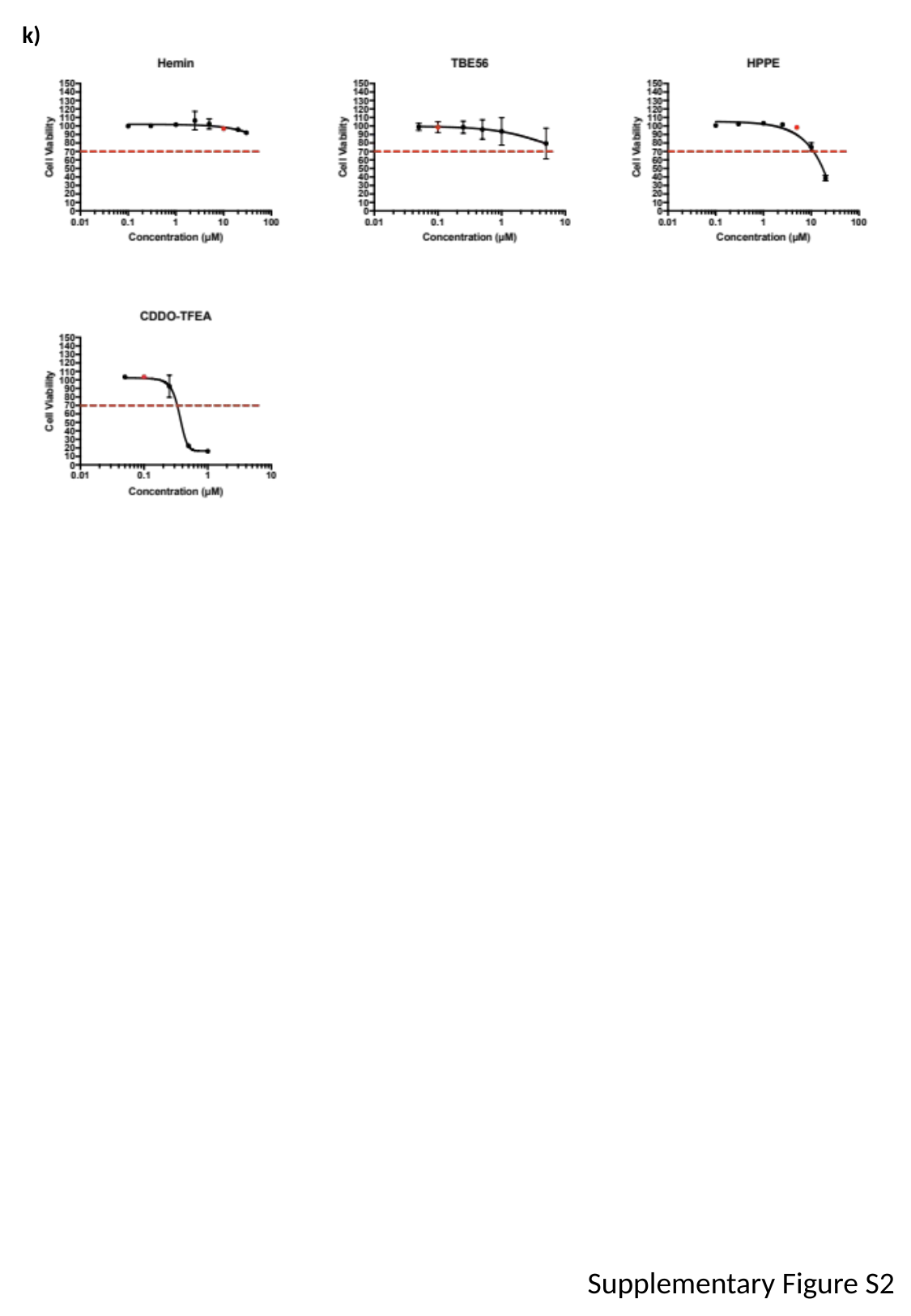

k)
Supplementary Figure S2

## Slide 10
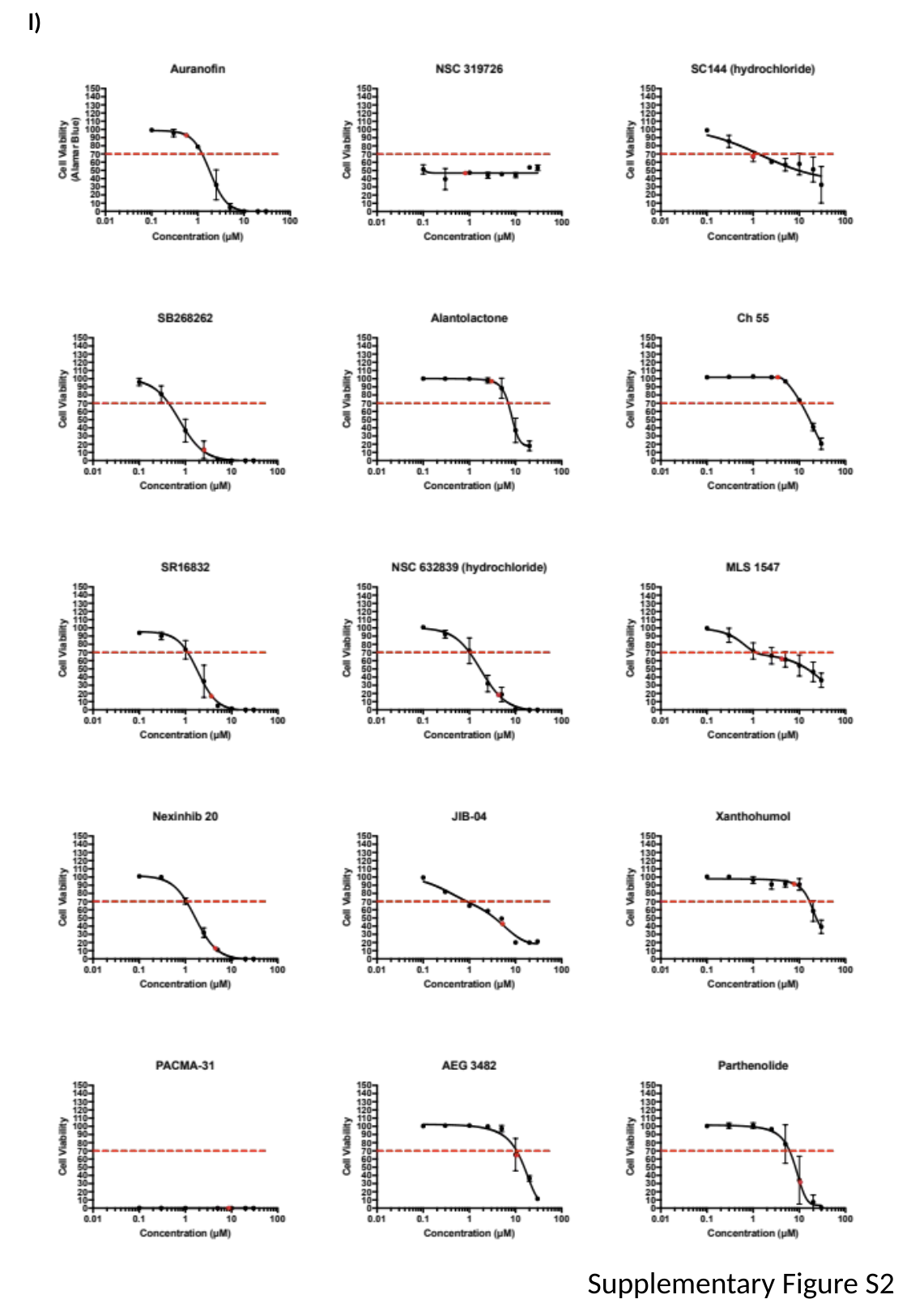

l)
Supplementary Figure S2

## Slide 11
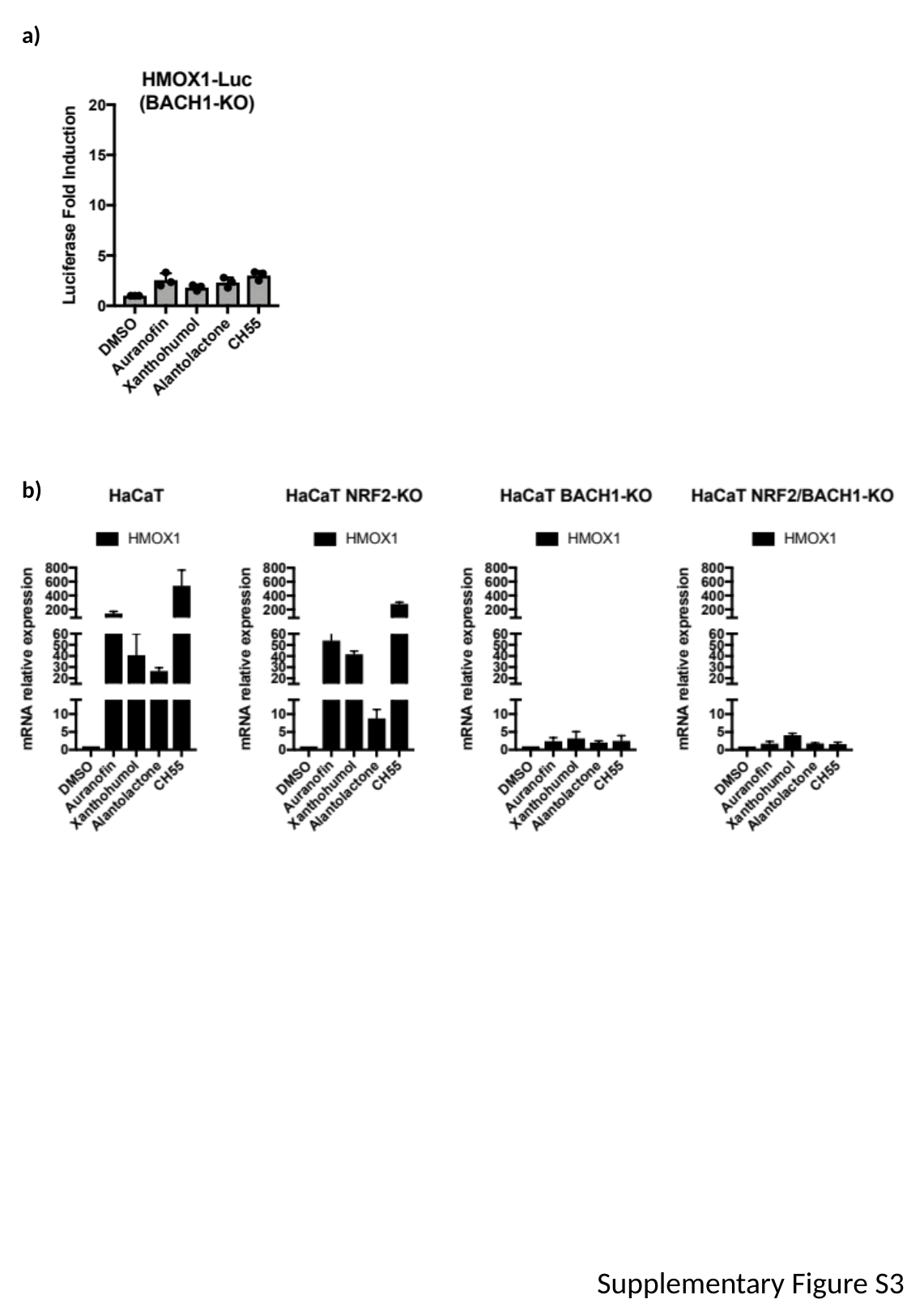

a)
b)
Supplementary Figure S3

## Slide 12
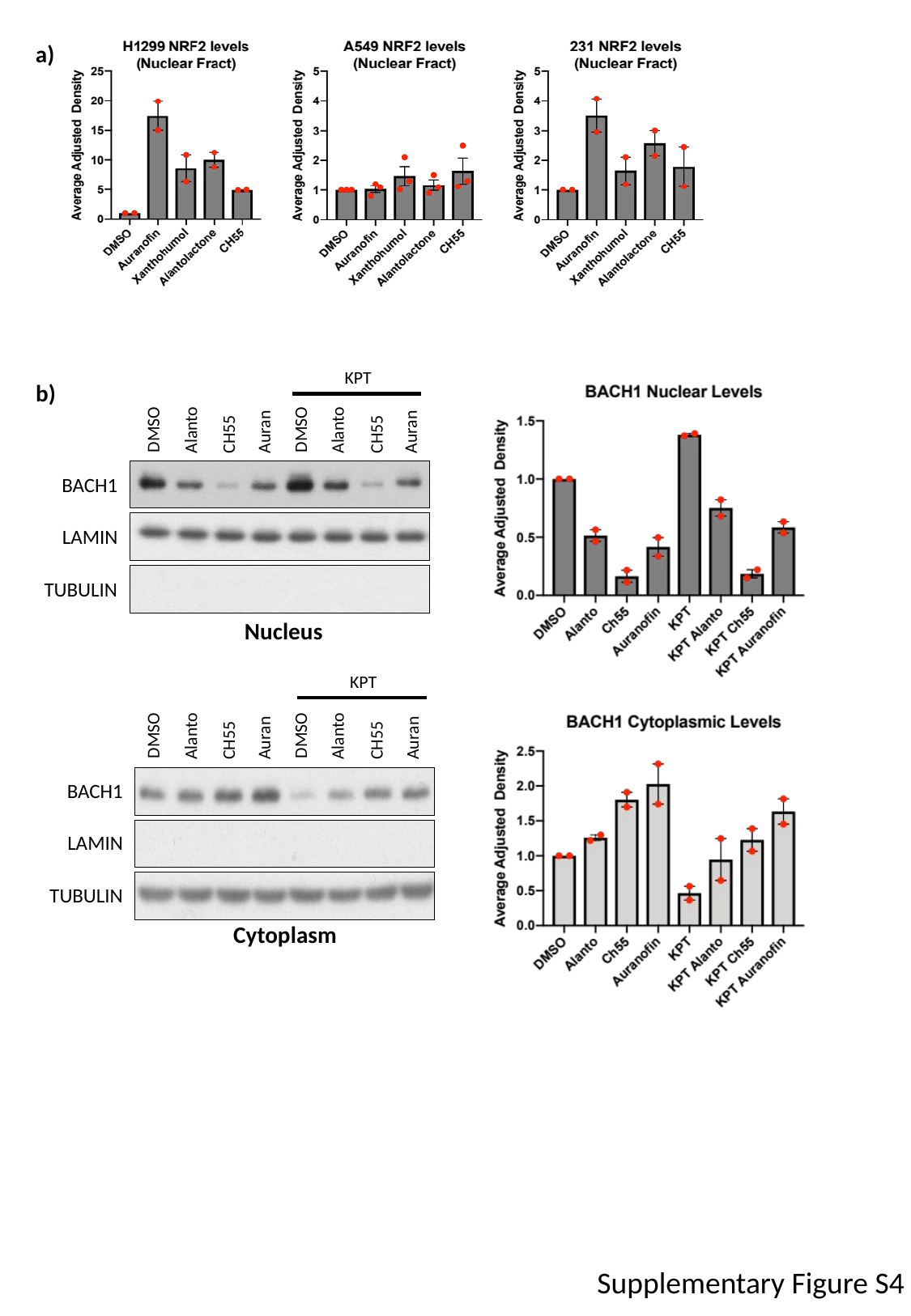

a)
KPT
DMSO
DMSO
Alanto
Alanto
Auran
Auran
CH55
CH55
BACH1
LAMIN
TUBULIN
Nucleus
KPT
DMSO
DMSO
Alanto
Alanto
Auran
Auran
CH55
CH55
BACH1
LAMIN
TUBULIN
Cytoplasm
b)
Supplementary Figure S4

## Slide 13
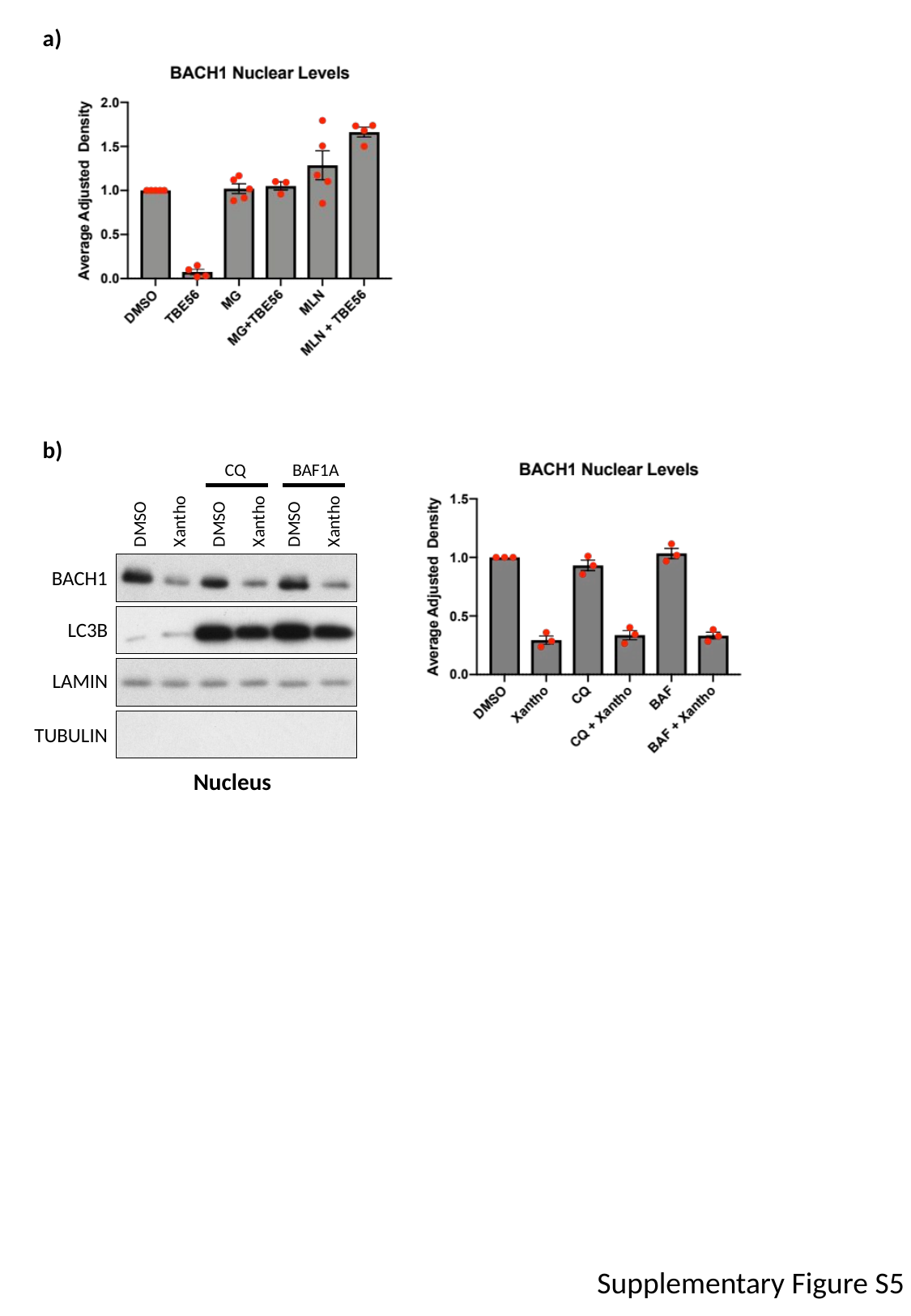

a)
b)
BAF1A
CQ
Xantho
Xantho
Xantho
DMSO
DMSO
DMSO
BACH1
LC3B
LAMIN
TUBULIN
Nucleus
Supplementary Figure S5

## Slide 14
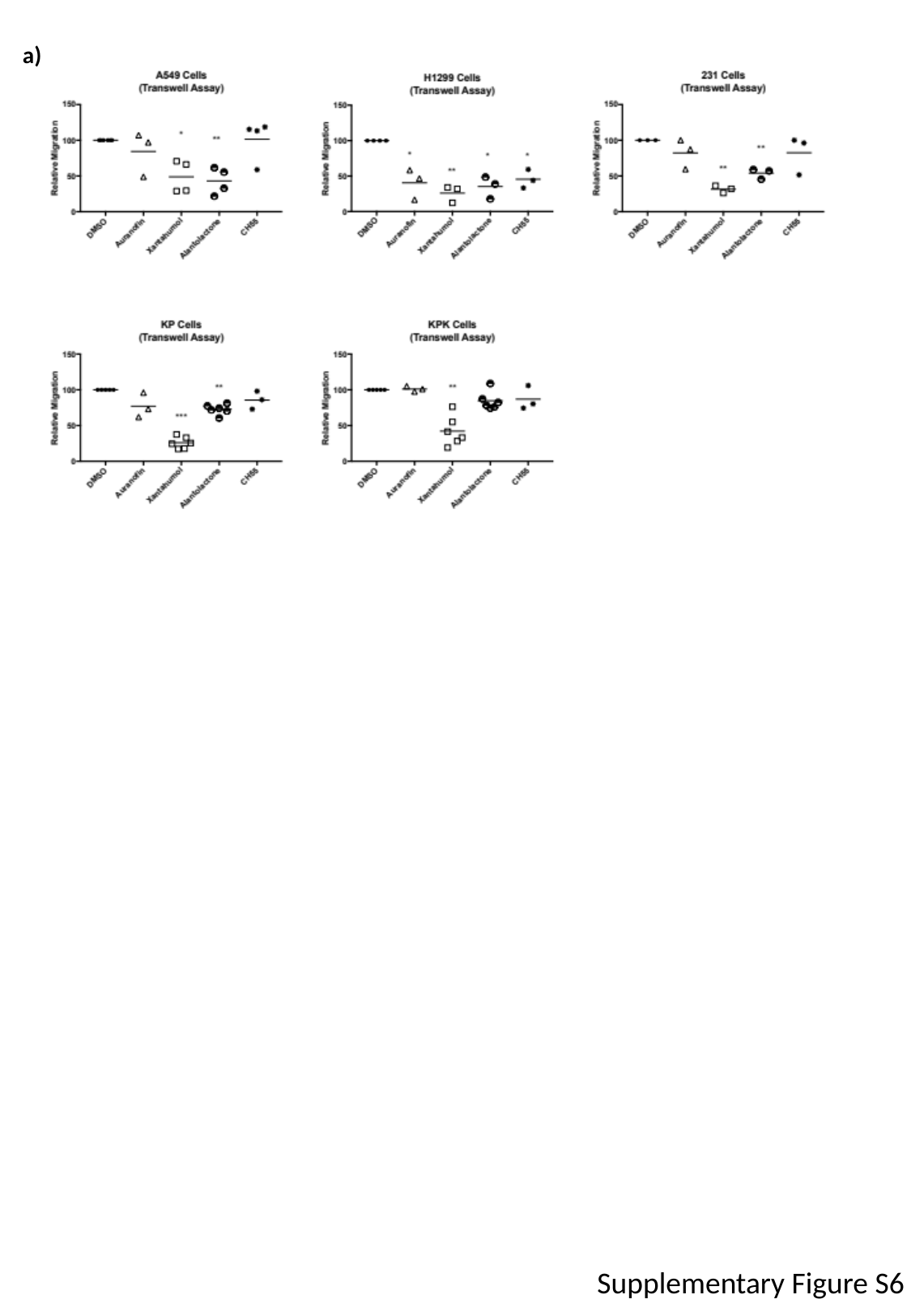

a)
Supplementary Figure S6
